# Long-term balancing selection contributes to adaptation in Arabidopsis and its relatives

**DOI:** 10.1186/s13059-017-1342-8

**Published:** 2017-11-15

**Authors:** Qiong Wu, Ting-Shen Han, Xi Chen, Jia-Fu Chen, Yu-Pan Zou, Zi-Wen Li, Yong-Chao Xu, Ya-Long Guo

**Affiliations:** 1 State Key Laboratory of Systematic and Evolutionary Botany, Institute of Botany, Chinese Academy of Sciences, Beijing, 100093 China; 2 University of Chinese Academy of Sciences, Beijing, 100049 China

**Keywords:** Adaptation, *Arabidopsis thaliana*, Balancing selection, *Capsella rubella*, Trans-species polymorphism

## Abstract

**Background:**

In contrast to positive selection, which reduces genetic variation by fixing beneficial alleles, balancing selection maintains genetic variation within a population or species and plays crucial roles in adaptation in diverse organisms. However, which genes, genome-wide, are under balancing selection and the extent to which these genes are involved in adaptation are largely unknown.

**Results:**

We performed a genome-wide scan for genes under balancing selection across two plant species, *Arabidopsis thaliana* and its relative *Capsella rubella*, which diverged about 8 million generations ago. Among hundreds of genes with shared coding-region polymorphisms, we find evidence for long-term balancing selection in five genes: AT1G35220, AT2G16570, AT4G29360, AT5G38460, and AT5G44000. These genes are involved in the response to biotic and abiotic stress and other fundamental biochemical processes. More intriguingly, for these genes, we detected significant ecological diversification between the two haplotype groups, suggesting that balancing selection has been very important for adaptation.

**Conclusions:**

Our results indicate that beyond the well-known S-locus genes and resistance genes, many loci are under balancing selection. These genes are mostly correlated with resistance to stress or other fundamental functions and likely play a more important role in adaptation to diverse habitats than previously thought.

**Electronic supplementary material:**

The online version of this article (doi:10.1186/s13059-017-1342-8) contains supplementary material, which is available to authorized users.

## Background

Understanding the maintenance of genetic variation in the face of genetic drift is of critical importance to decipher the mechanisms of adaptation. Balancing selection maintains advantageous genetic variation within a population [1–6] and has received long-term attention in evolutionary biology [7]. Classical examples are mainly restricted to the major histocompatibility locus (MHC) in vertebrates [8], ABO blood group in primates [5], heterokaryon incompatibility in fungi [9], and self-incompatibility (S) loci [10–13] and disease resistance (R) genes [14] in plants. Recently, a novel gene involved in resistance to severe malaria was found in a region of ancient balancing selection in a genome-wide association study [15]. In another case, balancing selection on the *srx-43* locus in *Caenorhabditis elegans* [16] surprisingly shapes density-dependent foraging behaviors. Obviously, almost all loci under balancing selection are important for the fitness of organisms.

Despite rapid progress in the understanding of balancing selection, confirmed cases are rather limited. Therefore, a question naturally follows, namely, what other genes in the genome are under balancing selection? The availability of genome-wide variation data provides the opportunity to comprehensively address this question.

**Fig. 1:**
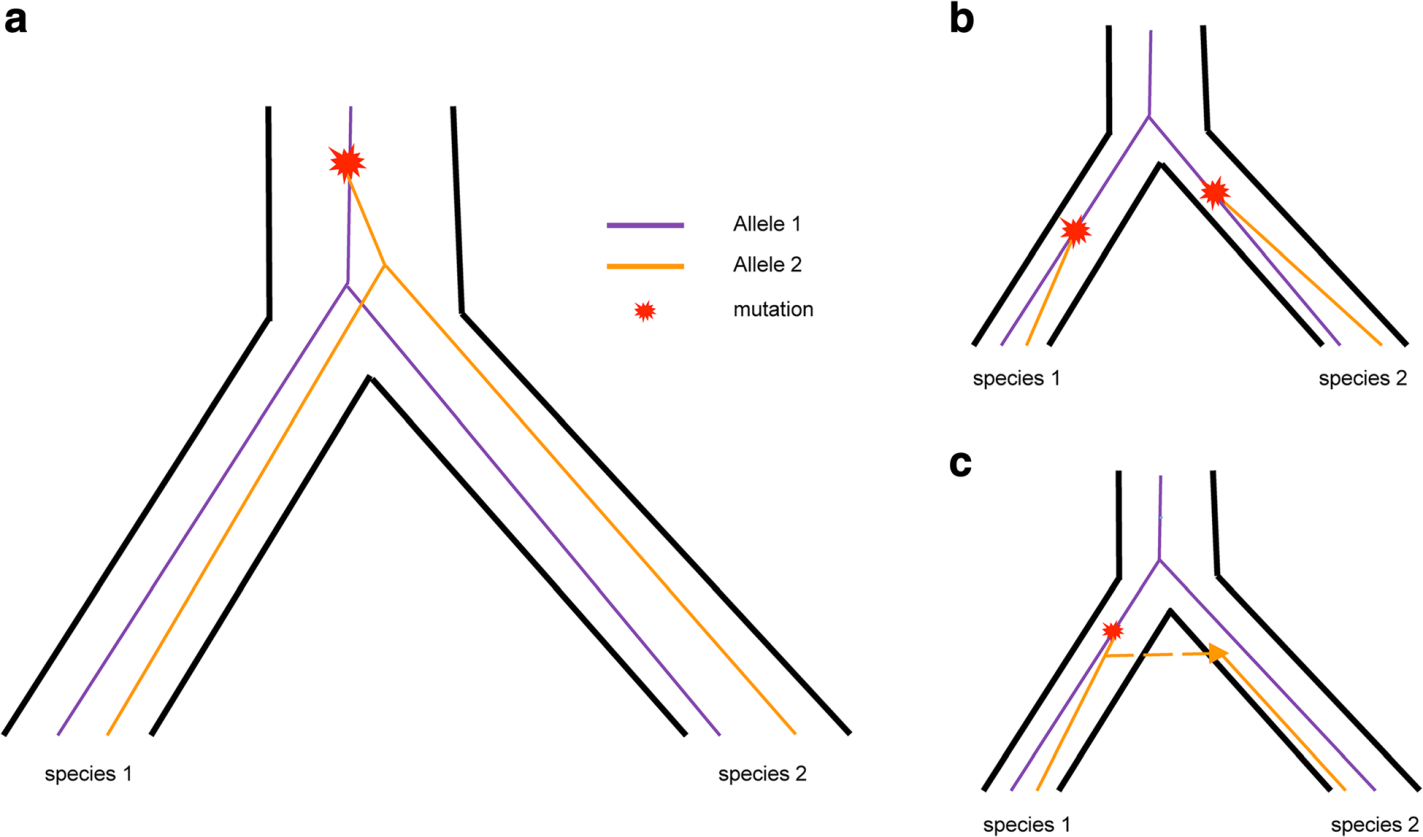
Schematic view of trans-species polymorphisms (**a**), recurrent mutations (**b**), and introgression events (**c**), all of which could give rise to shared polymorphisms

One convenient signature of ancient balancing selection is trans-species polymorphisms (TSPs) [2, 17], i.e. ancestral polymorphisms that arose before species diverged from a common ancestor, survived the split, and segregate in the present populations of different species. However, TSPs can be neutral if there is not enough time for genetic drift to erase the ancestral polymorphisms in any species. In contrast, if divergence time is sufficiently long, neutral TSPs will be eliminated and only the ones under balancing selection can survive. The surrounding ancestral regions of TSPs under balancing selection may be broken up by recombination, which becomes narrower as balancing selection becomes more ancient [5, 18]. Furthermore, TSPs can be easily confounded with recurrent mutations or introgression (Fig. 1), since both of the cases also produce shared polymorphisms in closely related species [17]. Nevertheless, some features are associated with ancient balancing selection [18]. In particular, orthologous sequences from different species cluster by allele, rather than species, due to tight linkage around the site under balancing selection [3, 18], while neutral recurrent shared polymorphisms still cluster by species. Another associated signal of long-term balancing selection is a high level of variation, since the polymorphisms surrounding TSPs should be more ancient than the genome-wide average coalescent time. Furthermore, the allele frequency distribution for sites under balancing selection is expected to exhibit a trend towards intermediate frequencies [18].

Genome-wide scans for TSPs are rather limited to Hominidae-related species [3, 19], except for one recent study in plants [20], in which a complex history of non-bifurcating speciation was identified in the genus *Arabidopsis* and many ancestral polymorphisms were detected. However, it is not clear whether these ancestral polymorphisms are maintained by balancing selection.

In this study, we performed a whole-genome scan for TSPs under balancing selection between two plant species, *Arabidopsis thaliana* and *Capsella rubella*. Unless otherwise specified, TSPs in this study refer to those under balancing selection, not neutral ones. *Arabidopsis* and *Capsella* belong to Brassicaceae and diverged about 8 million years (or ~ 8 million generations) ago [21], a much larger timescale than that of previous studies of humans and chimpanzees (~0.25 million generations) [3, 5, 19]. Beyond this, *C. rubella*, a highly selfing species, recently diverged from its outcrossing relative *Capsella grandiflora* and has experienced a severe population bottleneck, resulting in a tremendous reduction in genetic diversity and ancestral polymorphisms [22–24]. Therefore, TSPs that persist for such a long time, particularly through the dramatic demographic event, must be of critical importance for plants. We are particularly interested to see, as a kind of standing variation, the extent to which polymorphisms at loci under balancing selection could contribute to adaptation.

Interestingly, we detected 433 candidate genes that exhibit shared coding single nucleotide polymorphisms (SNPs) between the two species. We confirmed that five of these genes were under long-term balancing selection. Furthermore, there was significant ecological diversification between the two haplotype groups separated by the TSP sites. Our results indicate that, in plants, beyond the well-known S-locus genes and R genes, many loci are under balancing selection. These genes were mostly associated with resistance to stress and other fundamental functions and likely play an important role in adaptation to diverse habitats.

## Results

### Shared polymorphisms are abundant between *A. thaliana* and *C. rubella*

In a population of 80 *A. thaliana* accessions [25], there were 4,902,039 SNPs (out of 119,146,348 sites), among which 2,044,731 had a minor allele frequency (MAF) of > 0.05. In the *C. rubella* population, by calling SNPs from 22 *C. rubella* accessions (Additional file 1: Table S1, including 21 published accessions [26] and one accession sequenced in this study [27]) against the *C. rubella* reference genome [28], we identified 2,149,643 SNPs (out of 134,834,574 sites), of which 1,240,547 had a MAF > 0.05. To identify shared polymorphisms between the two species, defined as the same allele pair at a particular orthologous site, we first constructed the set of orthologous gene pairs between the two species. To guarantee that the orthologous genes are conserved, in addition to the reference genomes of *A. thaliana* and *C. rubella*, we included *Arabidopsis lyrata* [29], a congener of *A. thaliana*. We got 16,047 orthologous gene pairs and removed 33 that had tandem duplications in any of the three references and finally obtained a total of 16,014 orthologous gene pairs between *A. thaliana* and *C. rubella* for further analysis.

The genic region of the 16,014 orthologous genes in *A. thaliana* spanned 39,275,210 bp and similarly, in *C. rubella*, it spanned 40,936,262 bp. These regions contained 3,889,495 fixed differences and this high ratio (~ 10%) is consistent with the long divergence time (~ 8 MYA) of the two species [21]. In these regions, we found 1,122,845 bi-allelic sites (426,123 with MAF > 0.05) in *A. thaliana* and 452,116 bi-allelic sites (279,780 with MAF > 0.05) in *C. rubella*. Among these polymorphic sites, 19,732 orthologous sites were polymorphic in both species, of which 8535 shared the same allele pair (shared SNP [shSNP]) (Additional file 1: Table S2).

Compared with non-coding region sequences, coding region sequences are more conserved and yield robust alignments between the two highly diverged species; therefore, we first focused on shSNPs in coding regions. MAF > 0.05 was required in both species to guarantee SNP reliability and account for the expected excess of alleles with intermediate frequencies for sites under long-term balancing selection. We found 1503 shSNPs in the coding regions of 1007 genes.

**Fig. 2:**
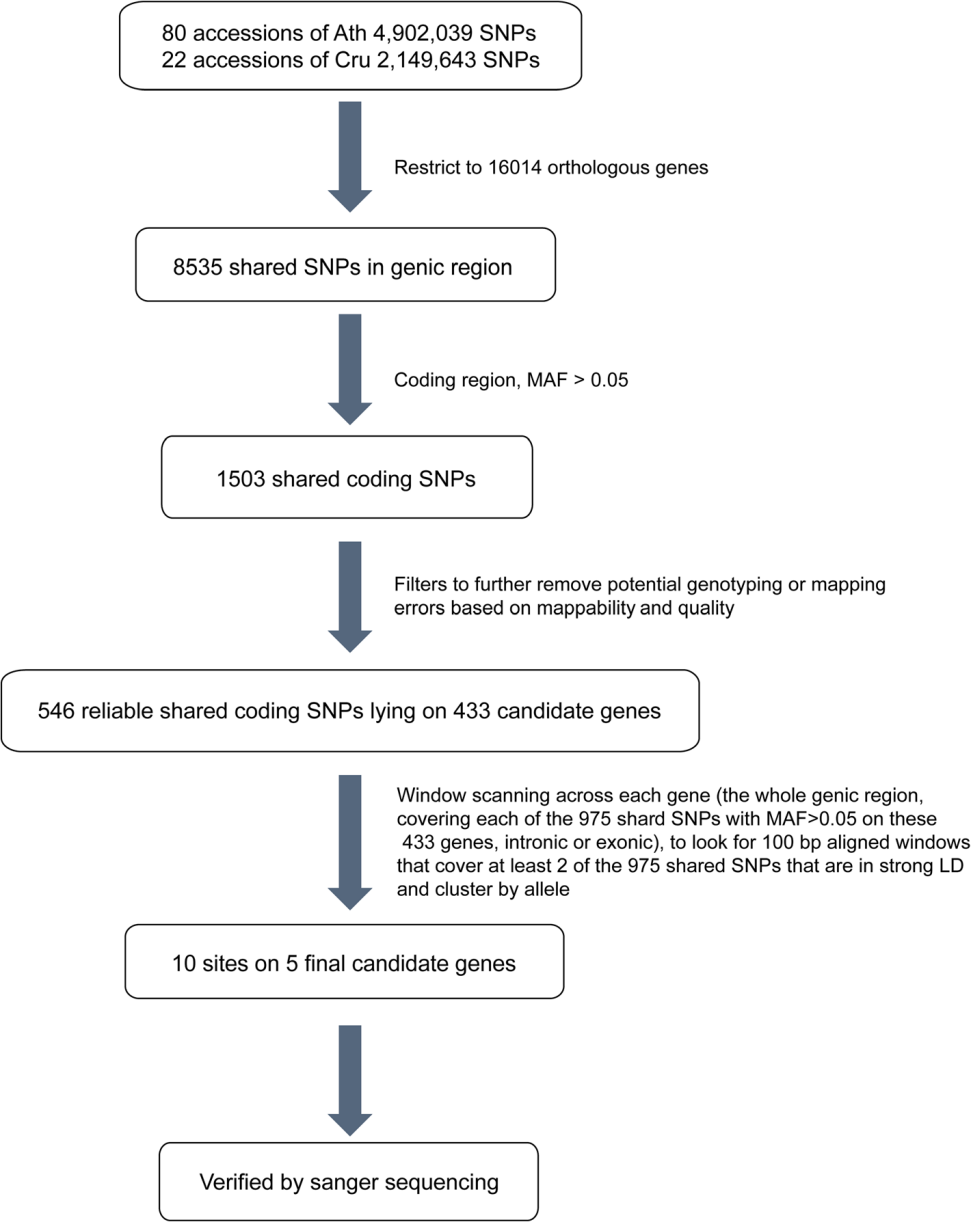
Pipeline of the SNP filtering process to identify candidate TSP sites

Further filtering was applied to the 1503 shSNPs to avoid genotyping and mapping errors. The filtering was only applied to the *C. rubella* SNP data, since we downloaded the SNP matrix for *A. thaliana*. To avoid spurious SNPs incurred by duplications in the genome, we assessed the mappability of every 50-bp region in *C. rubella* and only retained sites that were in uniquely mappable regions for subsequent analysis. This left only 580 sites. Finally, after removing low-quality sites marked by the SNP calling tool, we obtained 546 reliable shared coding SNPs in 433 genes. Details of the filtering process can be found in the “Methods” section and a view of the process is depicted in Fig. 2.

### Demographic history of the two species

The detection of real TSP signals from the abundant shared polymorphisms relies on a full understanding of the demographic history of the two species. The joint site frequency spectrum (joint SFS) has been widely used to study the demographic history of diverse organisms [30, 31]. Therefore, we first extracted the fourfold degenerate sites from the alignments of the reference genomes of *A. thaliana* and *C. rubella* on the 16,014 orthologues. Finally, we obtained 2,011,573 sites for the demographic analysis (see “Methods” for details).

**Fig. 3:**
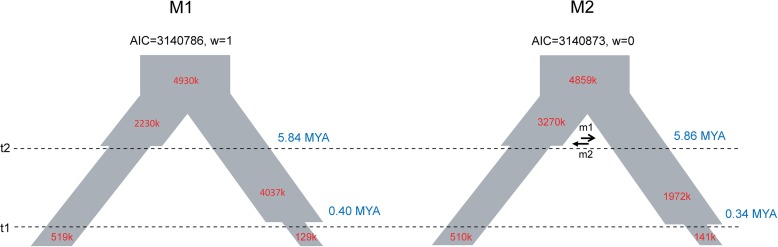
Demographic parameter estimates for two models of the divergence of the two species

Coalescence simulations were then run using fastsimcoal2 [30] under a basic model without gene flow (M1, Fig. 3) and a model incorporating ancient gene flow between the two genera (M2, Fig. 3). We considered only ancient gene flow between the two species, since species belonging to different genera and with different numbers of chromosomes (five vs eight) are highly unlikely to have recent introgression. In addition, in both genera, *A. thaliana* is the only species with five rather than eight chromosomes [32]; we therefore restricted the ancient gene flow before *A. thaliana* separated from the rest of the *Arabidopsis* genus. In each model, we set the divergence time of the two genera to 8 MYA [21], which amounts to 8 million generations ago, and assumed a spontaneous mutation rate of 7 × 10^–9^ per bp per generation [33]. We considered various population sizes for both species based on the transition events from their respective progenitors; *A. thaliana* underwent a population reduction after it diverged from the rest of the *Arabidopsis* genus around 6 MYA [21] and *C. rubella* experienced a very recent bottleneck associated with the speciation from *C. grandiflora* [23, 24]. We used coalescent simulations applying the composite likelihood method implemented in fastsimcoal2 [30] to fit both models to the joint SFS of the two species computed from the extracted 2,011,573 trans-species fourfold degenerate sites. We compared the two models using Akaike’s information criterion (AIC) and Akaike’s weight of evidence (w), as in Excoffier et al. [30]. The model without ancient gene flow (M1) fit slightly better (Max EstLhood: –682010 vs –682028), with a lower AIC and higher weight than those of the other model (Fig. 3, Additional file 2: Table S3). In addition, the two close likelihoods indicate that the effect of ancestral gene flow should have been wiped out over the long time scale and contributes little to model quality.

Under Model M1, the current *N*_e_ of *A. thaliana* was ~ 519,000 with a 95% confidence interval (CI) of 486,368–527,574, from a large ancestral population (~ 2,230,000, 95% CI = 1,085,330–4,876,051) before separating from the rest of the *Arabidopsis* genus at ~ 5.84 MYA (95% CI = 5.27–6.70). *C. rubella* evolved ~ 0.40 MYA (95% CI = 321,998–500,317) from an ancestral population with a large *N*_e_ of ~ 4,037,000 (95% CI = 2,076,868–5,165,614) and a current *N*_e_ of ~ 129,000 (95% CI = 126,383–157,779). The two genera diverged from an ancestral population with *N*_e_ = ~ 4,930,000 (95% CI = 4,560,931–4,969,696). Under Model M2 with gene flow, similar parameter estimates were obtained, except for a larger ancestral *N*_e_ for the *Arabidopsis* genus (~ 3,270,000, 95% CI = 797,016–4,342,346) and a smaller *N*_e_ for the *Capsella* genus (~1,972,000, 95% CI = 2,126,346–6,248,003). Stronger gene flow was estimated from *Capsella* to *Arabidopsis* than in the reverse direction (migration rate per generation; 1 × 10^–8^, 95% CI = 4.0 × 10^–15^–1.1 × 10^–6^ vs 7 × 10^–14^, 95% CI = 5.7 × 10^–15^–6.1 × 10^–5^), although both were weak (see Additional file 2: Table S3 for the details).

### Trans-species polymorphisms between the two species must be under balancing selection

Trans-species polymorphisms can be neutral and its probability can be approximated given specific demographic parameters. Similar to a study of TSPs in humans and chimpanzees [34], under neutral evolution, shared polymorphisms were identical by descent in our system only if: (1) at least two *A. thaliana* lineages and two *C. rubella* lineages did not coalesce before the *A. thaliana*–*C. rubella* split; and (2) lineages carrying the same allele coalesced before lineages carrying different alleles. This probability is mainly determined by condition (1) and can be approximated by the following based on the coalescent theory [34]:

$$ P={e}^{-\frac{T}{2{N}_A}\ast }{e}^{-\frac{T}{2{N}_C}}, $$

where *T* refers to the divergence time of the two genera and *N*_A_/*N*_C_ refers to the population sizes of *A. thaliana*/*C. rubella*, respectively. According to our estimates under Model M1, taking population size changes into consideration, this probability of identity by descent is on the order of 10^–9^. Given that we have < 39,275,210 aligned sites between the two species in genic region, we expect the total number of neutral TSPs to be < 1 by genetic drift alone.

We assumed random mating in our model; however, both species are selfing and population structure probably exists within species. Nevertheless, recent demographic events should have relatively little effect, since we require deep coalescence events by chance in both species in the same region of the genome [3, 18]. As illustrated in previous study [3], even deep population structure within modern humans should have minimal effect on the probability. In this study, both species have a history of predominantly outcrossing. *A. thaliana* transitioned from outcrossing to selfing only one million years ago [35] and *C. rubella* transitioned much more recently [23, 24]. Even as selfing species, the outcrossing rate of local populations is as high as 14.5% [36]. Therefore, population structures, if existing, are unlikely to persist over a long time scale and its impact on the probability can thus be ignored.

### Identification of trans-species polymorphisms under balancing selection

TSPs can be distinguished from neutral mutations because regions under long-term balancing selection cluster by allele, rather than by species [18]. Therefore, we next focused on the 433 candidate genes with reliable shared SNPs in the coding region and examined the haplotypes covering each shared bi-allelic SNP with MAF > 0.05 in the genic regions.

To estimate the length of each segment carrying a signal of TSPs, we used a formula derived previously [5] that relies largely on the recombination rate. From a coalescence point of view, such a segment is not broken up by recombination until all of the lineages from the same allelic class coalesce to their most recent common ancestor in the ancestral population [18]. Adopting a recombination rate of 3.6 cM/Mb [37] for both species, the length of the segment was extremely short, i.e. only several base pairs, theoretically. Given that both species recently arose from their respective outcrossing progenitors [23, 24, 35] and the effective recombination rate could be much higher in the past, the expected length may be even shorter. This estimate suggests, under the neutral circumstances in our system, that it is highly difficult to discover any segment without a break from recombination. However, when balancing selection exists, selection can suppress recombination in the surrounding region [34]. Therefore, the segment length should be longer than the theoretical estimated under a neutral model. We thereby scanned the genic region using a window size of 100 bp and a 1-bp step size.

In the 433 candidate genes, we detected 975 shared bi-allelic SNPs (including both exonic and intronic SNPs with MAF > 0.05). Similar to previous studies [3, 19, 20], we next looked for windows covering at least two of the 975 SNPs that are in strong linkage disequilibrium (*r* ^2^ > 0.5) in both species among the qualified windows (aligned at a minimum of 95% of the length; see “Methods” for details) to identify allelic trees. These restrictions can greatly reduce false positives and yield allelic trees, if they exist, with high resolution. Finally, we identified windows from five genes, AT1G35220, AT2G16570, AT4G29360, AT5G38460, and AT5G44000, involving ten sites, as candidate TSPs under long-term balancing selection (Additional file 3: Figure S1). None of the five orthologous genes we found here are correlated with copy number variation (CNV) and all of them have only one hit when we compared them against the references of the two species, respectively (see “Methods” for details).

**Fig. 4:**
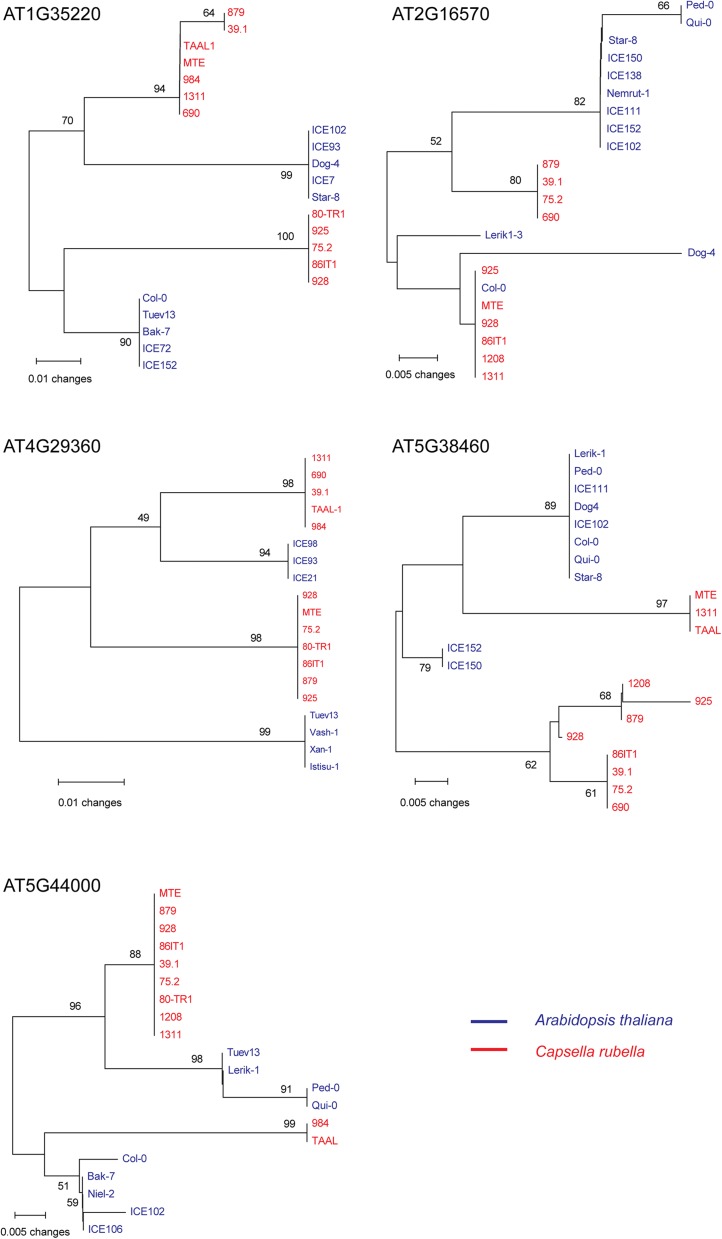
All candidate regions in the five genes produce an allelic tree, rather than a species tree

To verify the regions identified, we first determined all haplotypes in the identified regions from each population and resequenced representative accessions for each haplotype [38, 39] (see Additional file 1: Table S4 for the primers). As expected, all of the candidate TSP sites in the five genes were validated and the sequences of the two species in the candidate regions clustered by allele, rather than species (Fig. 4). In the gene AT1G35220, the two candidate TSP sites were in complete linkage disequilibrium in an intronic region; this region may be the target of balancing selection or linked to an undetected coding TSP site.

Although the haplotypes of each region clustered by allele, rather than species, haplotype sharing between the two species was rarely detected, except in AT2G16570 (Col-0 shared its haplotype with several *C. rubella* accessions; Fig. 4). This is not surprising given the long divergence time; extensive haplotype sharing usually appears at a much smaller timescale and is induced by events such as recent introgression between closely related species.

### Neutral simulation studies validate the five candidate genes

To see if the observed windows could be generated randomly under neutral evolution, resulting in false positives, we ran additional simulations based on the estimated demographic parameters using fastsimcoal2 [30] (Additional file 4: Text S1). Apart from neutral recurrent mutations, gene flow can also result in shared SNPs. Accordingly, we ran simulations under both Model M1 (without gene flow) and M2 (with ancient gene flow), although our demographic analysis indicated that M1 slightly better fit the data. In both simulations, we considered heterogeneity in mutation rates for different classes of mutations, notably the higher mutation rate at CpG sites, which may result in false positives (Additional file 1: Table S5, Additional file 4: Text S1). Using fastsimcoal2 [30], we generated 1,000,000 neutral segments of 100 bp under each model and looked for those with two or more shared SNPs and cluster by allele as we searched for TSPs.

For both models, none of the 1,000,000 runs gave rise to a window that met our criteria (Additional file 1: Table S6). Despite existence of neutral shared SNPs, no simulated window rendered an allelic tree, since all the windows with shared SNPs were accompanied by much more fixed differences between the two species, implying higher divergence levels than diversity. This result suggest that these simulated neutral shared SNPs are recurrent mutations, rather than TSPs, and more importantly, the five genes we found are not consistent with neutral evolution and thereby proved to be real TSPs under balancing selection. The final TSP sites and genes are listed in Table 1. Furthermore, together with the aforementioned demographic study, our results imply that even if ancient gene flow occurred, under neutral evolution, TSPs would be lost by drift in this system.

**Table 1:** Information on the candidate genes and TSP sites

Orthologous *A. th* gene	Orthologous *C. ru* gene	Positions in *A. th*	Positions in *C. ru*	SNP annotation	CpG status^a^	A1	A2	A2 frequency (*A. th*)	A2 frequency (*C. ru*)
AT1G35220	Carubv10011082m	Chr 1: 12913634	Scaffold 1: 12353061	Intron	No: TGC-TTC	G	T	46/81	5/23
		Chr 1: 12913648	Scaffold 1: 12353075	Intron	No: GAA-GGA	A	G	46/81	5/23
AT2G16570	Carubv10015505m	Chr 2: 7180661	Scaffold 3: 12816152	S	No: ACA-ATA	C	T	65/81	6/23
		Chr 2: 7180598	Scaffold 3: 12816089	S	Yes: TTG-TCG	T	C	66/81	6/23
AT4G29360	Carubv10007158m	Chr 4: 14452628	Scaffold 7: 4284494	S	Yes: CGG-CCG	C	G	5/81	13/23
		Chr 4: 14452661	Scaffold 7: 4284527	S	Yes: CGG-CAG	A	G	5/81	13/23
AT5G38460	Carubv10004565m	Chr 5: 15399653	Scaffold 7: 17116466	M	No: TTC-TGC	T	G	8/81	17/23
		Chr 5: 15399591	Scaffold 7: 17116528	S	No: TGC-TAC	G	A	7/81	14/23
AT5G44000	Carubv10026474m	Chr 5: 17702574	Scaffold 8: 1942064	S	No: TTA-TCA	T	C	75/81	4/23
		Chr 5: 17702547	Scaffold 8: 1942091	S	No: AGT-AAT	G	A	75/81	4/23

^a^Trinucleotides centered on the TSP sites are listed

### Properties of the genes under balancing selection

We next calculated nucleotide diversity (π) for all TSP regions in the five genes in each species and used the simulated neutral sequences under M1 to determine background diversity levels. All of the regions in the five genes exhibited significantly higher π values than background levels in both *C. rubella* and *A. thaliana* (Wilcoxon–Mann–Whitney test, FDR-corrected *P* < 0.05, Table 2, Additional file 3: Figure S2A), except AT5G38460 in *A. thaliana*. In addition, the alleles of these genes showed a trend towards intermediate frequencies (Wilcoxon–Mann–Whitney test, *P* = 0.0752/0.03474 for *A. thaliana*/*C. rubella*; Additional file 3: Figure S2B). However, an intermediate frequency is an indication of balancing selection, but not definitive evidence, since the allele frequency distribution of sites linked to a balanced polymorphism is expected to exhibit a shift towards the frequency equilibrium, which can be at any allele frequency [19].

**Table 2:** Genetic features of TSP sites

Orthologous gene (*A. th*)	Site label	Position in *A. th*	*r* ^2^ (*A. th*/*C. ru*)	π^*a*^ (*A. th/C. ru*)	MAF (*A. th*/*C. ru*)
AT1G35220	TSP-1	Chr 1: 12913634	1/1	0.036 (<2.2E-16)/0.037(<2.2E-16)	0.432/0.217
	TSP-2	Chr 1: 12913648			0.432/0.217
AT2G16570	TSP-3	Chr 2: 7180661	0.92/1	0.017 (2.9E-9)/0.019 (5.9E-10)	0.198/0.261
	TSP-4	Chr 2: 7180598			0.185/0.261
AT4G29360	TSP-5	Chr 4: 14452628	1/1	0.009 (1.8E-4)/0.034 (<2.2E-16)	0.062/0.434
	TSP-6	Chr 4: 14452661			0.062/0.434
AT5G38460	TSP-7	Chr 5: 15399653	0.55/0.86	0.007 (0.34)/0.031 (6.4E-3)	0.099/0.261
	TSP-8	Chr 5: 15399591			0.086/0.391
AT5G44000	TSP-9	Chr 5: 17702574	1/1	0.010 (1.4E-10)/0.023 (<2.2E-16)	0.074/0.174
	TSP-10	Chr 5: 17702547			0.074/0.174

^a^Values are the averages of the corresponding values in all 100 bp windows covering the TSP sites in strong linkage disequilibrium that could yield an allelic tree, and their associated FDR-adjusted *P* values are listed in parentheses

One of the five genes under long-term balancing selection in this study, AT1G35220, has an unknown function, but exhibits protein phosphorylation under ethylene treatment [40]. Among others, AT2G16570 is a key enzyme in the purine nucleotide biosynthesis pathway and is important for cell division, chloroplast biogenesis, and seed germination [41, 42]; AT4G29360 is an *O*-glycosyl hydrolase family 17 protein, involved in defense responses [43]; AT5G38460 is a glycosyltransferase and it catalyzes the transfer of a glycosyl group from one compound (donor) to another (acceptor) and is involved in diverse functions, including biotic stress [44]; AT5G44000 is a glutathione *S*-transferase, which is usually involved in the response to abiotic and biotic stress [45]. Apparently, these genes are potentially involved in the response to biotic or abiotic stress (AT4G29360, AT5G38460, and AT5G44000) or fundamental biochemical functions (AT2G16570).

As expected, the genes under balancing selection were functionally important and all of the homologues of the five genes already existed in the most recent common ancestor of green plants. As indicated in Table S7 (Additional file 1: Table S7), homologues (either orthologues or paralogues) can be found even in the most basal species of green plants, *Chlamydomonas reinhardtii*, for all of the five genes, except AT4G29360, which can be traced back to *Physcomitrella patens*.

However, loci that are widely accepted to be under balancing selection, such as the S-locus [10] or R genes [14], did not stand out in this study. This is expected, since these loci are too variable to identify based on short reads. For example, R genes are too dynamic to call SNPs [46]; the S-locus does not exist in the latest annotation of the *Arabidopsis* genome and only one S-locus haplotype is maintained in *C. rubella* since the transition from outcrossing to selfing and the breakdown of self-incompatibility [24]. Furthermore, the S-locus is no longer under balancing selection, since both species are now selfing. In contrast, the genes we identified here, although ancient, have not been comprehensively studied and may provide insight into the types of genes under balancing selection.

### Balancing selection contributed to adaptation to divergent habitats

To see if the allelic variants under long-term balancing selection are associated with ecological diversification, we investigated divergence with respect to 48 ecological factors (Additional file 5: Table S8A). Due to a lack of GPS information and the small sample size of *C. rubella*, this analysis was only possible for the *A. thaliana* samples. Population structure is usually highly correlated with ecological diversification and may therefore confound our results. We first checked whether any TSP site was correlated with population structure in the *A. thaliana* samples, although such structure does not affect the probability of observing the species tree of *A. thaliana* and *C. rubella*. Using ADMIXTURE [47], we found that the 80 *A. thaliana* samples can be classified into two groups (Additional file 3: Figure S3; Additional file 6: Table S9) and only the allelic classifications of the two sites from the gene AT5G38460 are significantly correlated with the population structure (chi-square test, FDR-corrected *P* < 0.05,; Additional file 1: Table S10). We thereby excluded AT5G38460 from subsequent ecological analyses.

To gain a thorough understanding of ecological divergence, we used 1135 recently published *A. thaliana* genomes [48]. First, we applied a “thinning” process to guarantee that every sample was highly representative of its natural habitat, which left 584 samples (see “Methods”). Second, for each gene, we classified the 584 accessions of *A. thaliana* into two groups based on the phased haplotypes for the two TSP sites (Additional file 5: Table S8B, C, some samples were removed because they could not be phased). We then evaluated divergence between the two groups of accessions with respect to the 48 ecological factors for each of the four genes. Interestingly, all of these four genes were associated with the divergence of some specific ecological parameters. AT1G35220 and AT4G29360, in particular, exhibited significant divergence with respect to most of the temperature-related ecological factors (Additional file 5: Table S8 A, Wilcoxon–Mann–Whitney test, FDR-corrected *P* < 0.05).

**Fig. 5:**
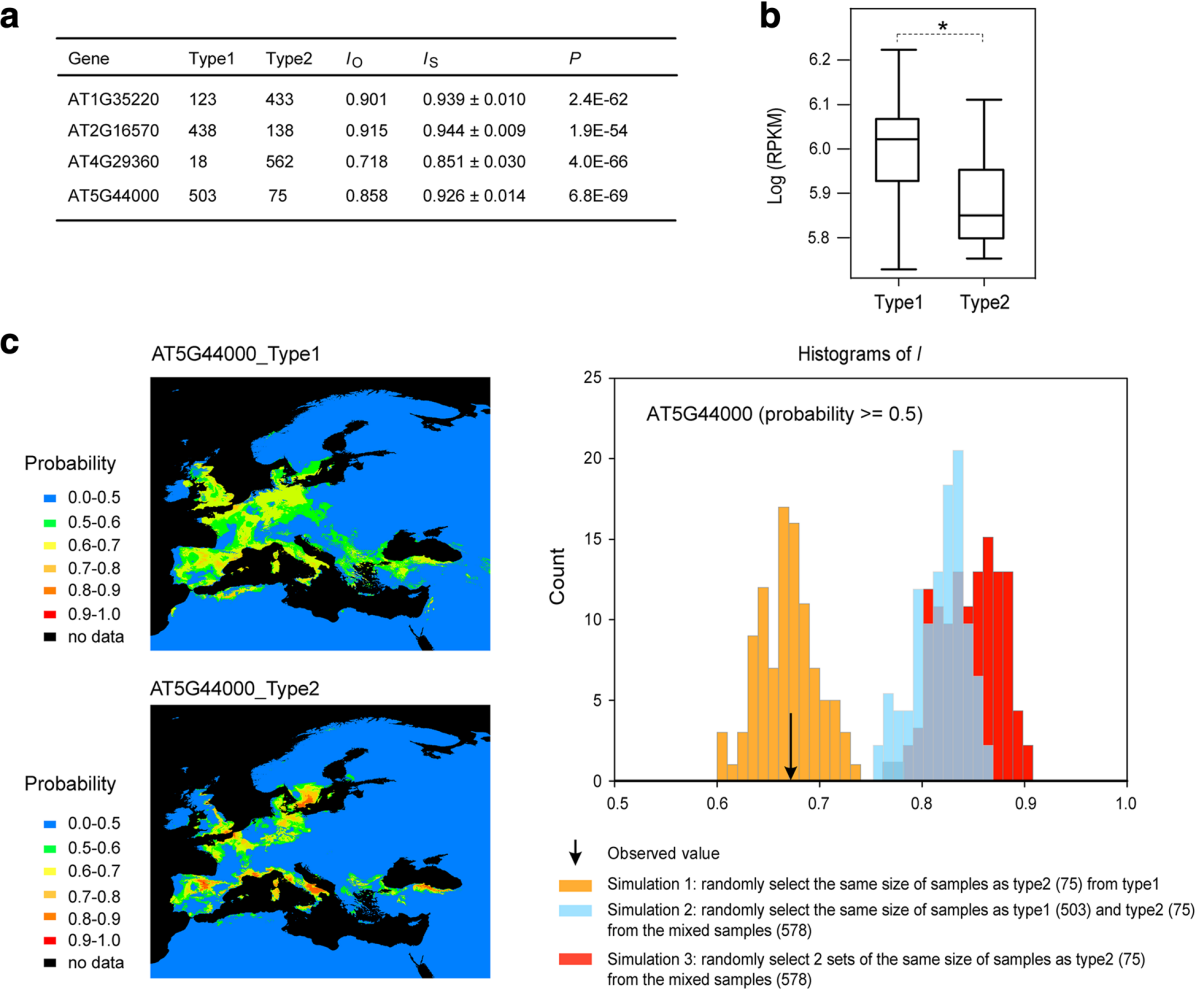
Ecological and expression divergence. **a** Significant ecological divergence between the two types of samples for each of the four genes, as indicated by the observed *I* score (*I*_O_) and simulated *I* scores (*I*_S_). **b** Expression divergence of the gene AT5G44000. **c** *Left*: Modeling of the niche with a high probability (≥ 0.5) of the two types of samples for AT5G44000. *Right*: Significance results under different permutation strategies (for niches with probability ≥ 0.5; *I*_O_ = 0.673, 100 permutations)

We next modeled the ecological niches for all four genes. Apparently, the two groups of samples for each gene, as indicated by Warren’s *I* statistics that measures niche similarity [49], exhibited significantly lower observed niche identity than 100 random permutations (one-sample *t*-test, FDR-corrected *P* < 0.01; Fig. 5a, Additional file 5: Table S8 D). In other words, the two allelic groups of samples exhibit significant niche divergence. Furthermore, the samples of each allelic type for each gene were scattered, instead of being isolated into a small local area (Additional file 3: Figure S4). These results suggest that all of these loci are correlated with adaptation.

We also examined expression differentiation for the four genes between the two corresponding groups based on the phased haplotypes at the two TSP sites choosing 84 published leaf-tissue-extracted transcriptomes from *A. thaliana* [50] (one sample was sequenced for each accession and the expression level was measured as fragments per kilobase of exon per million fragments mapped [FPKM]) as our previous study [51]. One gene, AT5G44000, exhibited significant expression difference (Wilcoxon–Mann–Whitney test, FDR-corrected *P* < 0.05, Fig. 5b) between the two haplotype groups.

We therefore performed in-depth niche modeling of AT5G44000 (Fig. 5c) and examined the diversification of the two groups of samples (503 vs 75). We first compared the niche identity between the two haplotype groups of AT5G44000 by restricting our analysis to niches with a high probability (≥ 0.5) and obtained similar results (Fig. 5c, Additional file 5: Table S8 D). To see if the unbalanced sample size could affect the results, we used another permutation strategy by restricting the analysis to the same sample size (75) for both sets in each repetition (with probability > 0.5). As presented in Fig. 5c, when the permutation was performed for the real sample groups (simulation 1), the observed *I* value (0.673) did not show a significant difference (one-sample *t*-test, *P* = 0.166), indicating that the observed value was reliable, regardless of the sample size difference. When the two real groups were mixed and two random groups of real sizes were selected (simulation 2) or two random groups of equal size (75) were selected (simulation 3), the difference between the observed value and the permutations was significant again (one-sample *t*-test, *P* = 1.9 × 10^–75^ for simulation 2 and *P* = 2.6 × 10^–75^ for simulation 3). These results imply that the two functionally differentiated haplotype groups of AT5G44000 adapted to divergent ecological habitats.

## Discussion

The importance of balancing selection for the maintenance of variation has received long-standing attention in evolutionary biology. However, many fundamental questions remain largely unanswered [3, 52]. For example, besides the well-known examples, what genes are under balancing selection across genomes, especially in plants? How long does genomic signature of balancing selection persist? In addition, as a kind of standing variation, to what extent does polymorphism of loci under balancing selection promote adaptation?

To address these questions, we performed a genomic scan for TSPs maintained in the model plant *A. thaliana* and its relative *C. rubella*, which diverged more than 8 million generations ago. TSPs can be neutral if the species diverged recently or have large effective population sizes. We therefore estimated demographic parameters for *A. thaliana* and *C. rubella* using population data and confirmed the theoretical prediction that neutral ancestral TSPs are highly unlikely in our system. After ruling out recurrent mutations and gene flow, we finally got five genes out of 433 candidate ones.

However, we focused on orthologous genes with two or more shared SNPs in both species, our method for TSP detection likely missed some genes with single TSP sites. Furthermore, we may have missed TSPs outside of genes; one previous study on humans and chimpanzees identified 123 non-coding TSPs and balancing selection has targeted regulatory variation in the human genome [3]. In addition, the further filters we used to get reliable shared coding SNPs may be too stringent, e.g. the mappability filter removed two-thirds of the shared SNPs. Nevertheless, this is a reasonable strategy to avoid false positives.

The genes under balancing selection may have played critical roles in adaptation to divergent habitats, since for four of the genes under balancing selection, the niches of the two haplotype groups were significantly differentiated. Unlike animals, in which genes experiencing balancing selection are mainly involved in resistance to viruses, in this study we found some other genes under balancing selection in plants, correlated with the response to stress and fundamental functions. In particular, these genes were correlated with diversifying niches, indicating that balancing selection probably has more influences on ecological distribution in plants than in animals. However, we cannot exclude the possibility that other environmental factors, especially microhabitat or biotic factors, like pathogens, contributed to the maintenance and spread of the genetic footprint of balancing selection [6]. More correlations may be discovered if additional factors are considered.

Loci under balancing selection are a good system to understand the mechanism of adaptation. The identification of genetic loci associated with both particular climatic factors and fitness traits is critical for the understanding of adaptive mechanisms in organisms, but this is extremely difficult to achieve given the limited time and usually weak selection strength [53, 54]. However, balancing selection can act through a variety of mechanisms [1, 2]. For example, by temporal and spatial variation in selection [55], loci can be functionally associated with local or global environmental changes during their long-term evolutionary history. In this way, strong and clear selection signal could be kept in the genome and could be studied in-depth. For example, a recent study identified a novel locus of resistance to severe malaria [15], which was in a region of ancient balancing selection [3].

Finally, it will be interesting to determine how genetic differentiation at these loci contributes to adaptation to different habitats. Given that *C. rubella* experienced a severe population bottleneck during the breeding system transition [23, 24], TSPs that survived this dramatic demographic event could be of critical importance for plant fitness. Therefore, it will be highly rewarding to further investigate the biological functions of these five genes under ancient balancing selection or genes with candidate TSPs to determine the biological basis for balancing selection. Moreover, a genome scan for loci under balancing selection is an effective way to reveal functionally important genes or sequences that are difficult to detect using common reverse or forward genetics owing to weak or indirect effects on organisms.

## Conclusions

We identified a number of shared polymorphic sites in the genomes of two closely related species and confirmed that at least five genes are under long-term balancing selection. The two different haplotypes of these genes under balancing selection were significantly differentiated with respect to habitats, suggesting that balancing selection could have contributed to adaptation to different habitats in plants in general.

## Methods

### Species sampling and DNA sequencing

The SNP matrix of 80 accessions of *A. thaliana* genomes (with ecotype Col-0 as the reference) [25] was downloaded from the *Arabidopsis* 1001 Genome Database (http://1001genomes.org/) and genome sequences of 21 *C. rubella* accessions were downloaded [26]. Together with another accession (928) sequenced here [27] and the reference accession MTE, 23 samples comprised the *C. rubella* population (see Additional file 1: Table S1 for a complete list of the *C. rubella* samples). Genomic DNA of accession 928 was extracted from seedlings using the CTAB method [56]. Paired-end sequencing libraries with an insert size of approximately 300 bp were constructed. Then, 100-bp pair-end reads were sequenced using Illumina HiSeq 2000. To verify the sequences, partial fragments of the five candidate genes from representative ecotypes of *C. rubella* and *A. thaliana* were amplified and sequenced [38, 39]. Primers used for PCR and sequencing are listed in Additional file 1: Table S4. PCR products were sequenced using the ABI 3730 automated sequencer (Applied Biosystems, Foster City, CA, USA).

### Read processing, mapping, and genotyping

Raw short reads were filtered using NGSQCToolkit v2.3.3 [57] with a cutoff PHRED quality score of 20. The percentage of qualified nucleotide bases in a read should not be < 70%. Every *C. rubella* sample was aligned to the reference MTE genome using BWA v0.7.12 [58] with default parameters allowing up to 4% mismatches and one gap. The reads were sorted, and duplicates were removed using the toolkit Picard v2.0.1 (http://broadinstitute.github.io/picard/). The rest of the genotyping pipeline was performed using the toolkit GATK v3.5 [59]. Reads present in areas surrounding InDels were realigned using the built-in function IndelRealigner, after which SNPs were called using UnifiedGenotyper. Finally, a series of quality filters were applied to reduce systematic errors, including mapping quality (MQ) ≥ 20, quality-by-depth ratio (QD) ≥ 2, ReadPosRankSum ≥ –8.0, depth coverage (DP) ≥ 3, probability of strand bias (FS) ≤ 30.0, and no more than three SNPs within 10 bp. SNPs that passed these filters were kept for subsequent analyses.

### Identification of orthologues and homologues

Orthologous gene groups in *A. thaliana*, *A. lyrata*, and *C. rubella* were identified using InParanoid v2.0 [60] with default parameters. MCScanX [61] was used to identify synteny in the three genomes to confirm the orthologous relationships of these genes. Only those gene groups identified by InParanoid and supported as syntenic were considered orthologous. To assess the number of homologues for the five candidate genes with TSP sites, each gene was searched against the representative genomes of 11 green plants using blastp with an *E*-value threshold of 1 × 10^–5^ according to our previous study [62] (Additional file 1: Table S7).

### Identification of shared SNPs

The sequences of all samples for each orthologous gene were aligned using MUSCLE v3.8.31 [63]. Shared bi-allelic SNPs were counted. The process was repeated for both coding regions and genic regions.

To guarantee the reliability of shared SNPs, further filtering steps were applied to the *C. rubella* SNPs. The mappability of each 50-bp region in the reference genome of *C. rubella* was assessed using the 50-bp mappability score [64], which counts the number of times each 50 mer maps to the reference *C. rubella* genome, allowing up to two mismatches. SNPs any of whose overlapping 50 mers could be mapped to more than one locations with two or fewer mismatches were excluded.

To determine whether candidate genes fell in regions of CNV, CNVs were detected in each species using CNVnator [65] with a bin size of 500 bp and a *P* value threshold of 0.01. Candidates with normalized read depths < 0.5 or > 1.5 were considered as CNVs of deletion or duplication events, respectively. snpEff v4.2 [66] was used to annotate the variation for each sample, and SNPs were characterized as synonymous_variant (S), missense_variant (M), or intron_variant (intron).

### Demographic inference

To uncover the neutral evolutionary history of the two species, the orthologous fourfold degenerate sites were extracted from the alignments of the respective reference genomes of *A. thaliana* and *C. rubella* for the coding regions of orthologues. The three codons of an orthologous amino acid from the two species were required to be aligned together (i.e. not separated by gaps). Afterwards, by restricting the analysis to fixed or bi-allelic sites, 2,011,573 orthologous fourfold degenerate sites were finally used to generate the joint SFS of the two species under study. Fastsimcoal2 [30] was then run on the SFS under Model M1 (no gene flow) and M2 (ancient gene flow). The best parameter estimates under each model were obtained from 50 independent runs with a minimum of 100,000 and a maximum of 1,000,000 coalescent simulations as well as 10–40 cycles of the likelihood maximization algorithm. SFS entries with support from fewer than six SNPs were ignored, as suggested by Excoffier et al. [30]. The 95% CIs of the best parameter estimates were obtained by non-parametric block bootstrapping, as in Malaspinas et al. [67], and each orthologue was considered as a block.

### Allelic tree searching

A sliding window approach was used to scan every valid 100-bp window that covered each shared SNP in the genic regions of the 433 candidate genes with reliable shared coding SNPs for regions of sequences clustering by allele. To be a valid window, the number of effectively aligned sites (A, C, G, or T) must be no less than 95 in each species to minimize gaps or missing bases in the *A. thaliana* SNP matrix. Phylogenetic neighbor-joining tree [68] construction was performed using the Phylip package with default parameters [69] and each tree was checked whether all the samples from one species were clustered together against the other. Trees that failed such clustering became candidate allelic trees and were checked manually, topological robustness was evaluated with 1000 bootstrapping replicates using MEGA v6 [70]. Linkage disequilibrium was evaluated by calculating *r* ^2^ using PLINK [71].

### Analysis of PCR-verified candidate regions

All haplotypes for each candidate region were obtained using DnaSP v5.10.1 [72]. Neighbor-joining trees of the PCR sequences of the candidate regions under balancing selection were constructed using MEGA v6 [70] with the Kimura two-parameter model [68, 73]. Topological robustness was assessed by bootstrapping with 1000 replicates [74].

### Ecological analysis

ADMIXTURE [47] was used to estimate the population structure in the 80 *A. thaliana* samples [25]. Such analysis was run based on the 1,438,787 SNPs removing sites ambiguous in any sample from the original SNP matrix; group numbers (*K*) from 1 to 10 were explored. Finally, *K* = 2, with the lowest cross-validation error, was the fittest number. Correlations between the structure groups and allelic types were assessed by chi-square tests.

To reduce the effects of sampling bias, sample records were spatially thinned using the randomization method in the spThin R package [75]. The “thin” method was used, setting the parameter “thin.par” to be 5 km, namely, every two samples should be at least 5 km apart.

To investigate the ecological characteristics associated with the loci under ancient balancing selection, ecological niche modeling (ENM) and ecological niche identity tests were performed [49]. ENMTools [76] was used to calculate the niche overlap between the two haplotype groups assessed by Warren’s *I* similarity statistics, ranging from 0 (no overlap) to 1 (identical niches), with 100 pseudo-replicates, according to our previous study [77]. The one-sample *t*-test was employed when comparing the observed *I* score with the scores for a set of permutations. Different permutation strategies were achieved by modifying the options in ENMTools [76].

### Statistical analysis

All statistical analyses were performed using R package v3.1.3 [78]. All *P* values from multiple testing were adjusted using the “fdr” option in the “*p.adjust*” function in R [79]. All violin plots and histograms were generated using R as well.

## Additional files

Additional file 1: Table S1. List of *C. rubella* accessions included in this study. **Table S2.** Summary of SNPs called from populations of each species. **Table S4.** Primers for PCR amplification and sequencing. **Table S5.** Statistics of the SNPs at CpG and non-CpG sites in the genic regions of 16,014 orthologous genes. **Table S6.** Simulation results for different demographic models. **Table S7.** Number of homologous genes for each of the confirmed genes with trans-species polymorphism signals in green plants. **Table S10.** Correlation between the structure and allelic type in 80 *A. thaliana* samples for each of the five genes under balancing selection. (DOCX 63 kb)

Additional file 2: Table S3. Demographic inference results from fastsimcoal2. (XLSX 15 kb)

Additional file 3: Figure S1. Allelic trees across the two species based on the 100-bp window around the TSP sites for each of the five genes under balancing selection. All *A. thaliana* accessions are colored in *red* and numbered according to the accessions listed on the 1001 Genomes site (http://1001genomes.org/projects/MPICao2010/index.html); see Additional file 5: Table S8C for details. All *C. rubella* accessions are shown in *black* and numbered according to Additional file 1: Table S1. **Figure S2.** Distribution of the (**A**) nucleotide diversity (π) and (**B**) MAF values of the simulated neutral sequences of 100 bp under the estimated model in each species. *Triangles* in different *colors* in (**A**) indicate the average values for all qualified windows in the five genes. See Table 1 for the details of each site (labeled TSP-1 to TSP-10). **Figure S3.** Cross-validation errors for various numbers of clusters (*K*) in an ADMIXTURE analysis. **Figure S4.** Geographic distributions of samples of different allelic types for the four genes under long-term balancing selection excluding AT5G38460. (DOCX 5905 kb)

Additional file 4: Text S1. Coalescence-based simulation to support the candidate TSPs. (DOCX 45 kb)

Additional file 5: Table S8. Ecological analysis: source data and results. A. The 48 ecological factors used in the analysis and the divergence of the two types of samples for each gene with regard to these factors. In each column of the four genes, the FDR-corrected *P* values are listed, and the significant ones are highlighted in red. B. Sample size distribution based on the haplotype phasing for each gene before and after thinning of the 1135 genomes. C. Sample information and classification for each gene. “-” indicates that the corresponding sample is not phased properly in the corresponding gene. D. The ecological significance of the four genes. (XLSX 52 kb)

Additional file 6: Table S9. Structure of the 80 *A. thaliana* samples. The values for Group 1 and Group 2 indicate the corresponding ancestral fraction, respectively. (XLSX 12 kb)
