# Supplementary material for: Long-term balancing selection contributes to adaptation in Arabidopsis and its relatives
Source: Genome Biol. 2017 Nov 15;18:217. doi: 10.1186/s13059-017-1342-8 (PMC5686891; doi:10.1186/s13059-017-1342-8)
Supplement: Supplementary file 1 — List of C. rubella accessions included in this study. Table S2. Summary of SNPs called from populations of each species. Table S4. Primers for PCR amplification and sequencing. Table S5. Statistics of the SNPs at CpG and non-CpG sites in the genic regions of 16,014 orthologous genes. Table S6. Simulation results for different demographic models. Table S7. Number of homologous genes for each of the confirmed genes with trans-species polymorphism signals in green plants. Table S10. Correlation between the structure and allelic type in 80 A. thaliana samples for each of the five genes under balancing selection. (DOCX 63 kb) [file 13059_2017_1342_MOESM1_ESM.docx]

**Additional file Tables for**

**Long-term balancing selection contributes to adaptation in *Arabidopsis* and its relatives**

Qiong Wu^1^, Ting-Shen Han^1,2^, Xi Chen^1,2^, Jia-Fu Chen^1,2^, Yu-Pan Zou^1,2^, Zi-Wen Li^1^, Yong-Chao Xu^1,2^, Ya-Long Guo^1,2*^

^1^ State Key Laboratory of Systematic and Evolutionary Botany, Institute of Botany, Chinese Academy of Sciences, Beijing 100093, China

^2^ University of Chinese Academy of Sciences, Beijing 100049, China

* Corresponding author: Ya-Long Guo

State Key Laboratory of Systematic and Evolutionary Botany

Institute of Botany, Chinese Academy of Sciences

Beijing 100093, China

PH +86-62836298; FX +86-62590843

EM yalong.guo@ibcas.ac.cn

Running title: Balancing selection shapes evolution of *Arabidopsis* relatives

Table S1. List of *C. rubella* accessions included in this study.

| No. | Accession name | Reads left after filtering (%) | Number of SNPs (in thousands) | Reference |
| --- | --- | --- | --- | --- |
| 1 | 100.8 | 67.64 | 606 | [[10](#_ENREF_10)] |
| 2 | 1208 | 71.84 | 405 |  |
| 3 | 1245-12 | 68.81 | 405 |  |
| 4 | 1249-11 | 64.77 | 386 |  |
| 5 | 1267-15 | 67.15 | 403 |  |
| 6 | 1311 | 67.76 | 368 |  |
| 7 | 1316-5 | 62.96 | 375 |  |
| 8 | 1321-1 | 61.86 | 379 |  |
| 9 | 1354-12 | 70.75 | 305 |  |
| 10 | 1575-1 | 53.76 | 366 |  |
| 11 | 39.1 | 60.58 | 443 |  |
| 12 | 690 | 73.6 | 401 |  |
| 13 | 75.2 | 57.56 | 579 |  |
| 14 | 79.1 | 71.03 | 550 |  |
| 15 | 80TR1-TS1 | 68.5 | 524 |  |
| 16 | 86IT1 | 46.63 | 363 |  |
| 17 | 879 | 64.49 | 620 |  |
| 18 | 925 | 66.87 | 586 |  |
| 19 | 984 | 47.17 | 373 |  |
| 20 | 987-25 | 60.71 | 395 |  |
| 21 | TAAL-1 | 71.5 | 378 |  |
| 22 | 928 | 92.82 | 335 | this study |

Table S2. Summary of SNPs called from populations of each species.

| Site type | *A. thaliana* | *C. rubella* |
| --- | --- | --- |
| whole genome | | |
| reference sites | 119,146,348 | 134,834,574 |
| SNPs | 4,902,039 | 2,149,643 |
| non-singleton SNPS | 3,094,349 | 1,240,547 |
| SNPs (MAF >0.05) | 2,044,731 | 1,240,547 |
| 16,014 orthologous genes | | |
| reference sites | 39,275,210 | 40,936,262 |
| bi-allelic SNPs | 1,122,845 | 452,116 |
| bi-allelic SNPs (MAF >0.05) | 426,123 | 279,780 |
| fixed differences | 3,889,495 | |
| orthologous (bi-allelic) SNPs in both species | 19,732 | |
| shared (bi-allelic) SNPs | 8,535 | |
| shared coding (bi-allelic) SNPs (MAF >0.05) | 1,503 | |
| final reliable shared coding (bi-allelic) SNPs | 546 | |

Table S4. Primers for PCR amplification and sequencing.

| Genes | Primer sequences | Primer positions |
| --- | --- | --- |
| AT1G35220  Carubv10011082m | CAAGCATGATCTTGATAGCT  TTAGTGAAACCGAAATAACC  GTTCCATTCCTCATTTCG  CAACACACTTTTCATCCC | Chr_1: 12912938–12912957  Chr_1: 12914060–12914079  Scaffold_1: 12352435–12352452  Scaffold_1: 12353707–12353724 |
| AT2G16570  Carubv10015505m | GGTCGGTCCGTGTATGAATC  CATCAATGAAATCCCCTCCT  CGACTTCGTCTAAGATTGTTC  CCTCGTTTCACTTTTACCTC | Chr_2: 7181079–7181098  Chr_2: 7180474–7180493  Scaffold_3: 12816268–12816288  Scaffold_3: 12815985–12816004 |
| AT4G29360  Carubv10007158m | ATGCTTGAGTTCTTGGTTGAG  CGAGTTACCGTTGATAATTGGA  ATGCTTGAGTTCTTGGTTGAG  CGAGTTACCGTTGTTAATTGGA | Chr_4: 14452870–14452850  Chr_4: 14452220–14452241  Scaffold_7: 4284252–4284272  Scaffold_7: 4284915–4284894 |
| AT5G38460  Carubv10004565m | TTCTCACTTGTGTCTTGTTC  AAGAGAGCGTAATAGGTTAA  TTGATTGTGTCCATGTTGCA  TTTGAGGGATGGTGTTGTGA | Chr_5: 15400124–15400143  Chr_5: 15398962–15398981  Scaffold_7: 17116370–17116389  Scaffold_7: 17119828–17119849 |
| AT5G44000  Carubv10026474m | AAGAGATGATACAAGGTTGG  GTATGATACTTTTGAGTGCTGT  TACGAGGGGAGATGTACGGT  TTAATGGTGCAGAAGGCAAG | Chr_5: 17703212–17703231  Chr_5: 17702169–17702190  Scaffold_8: 1941205–1941224  Scaffold_8: 1942871–1942890 |

Table S5. Statistics of the SNPs at CpG and non-CpG sites in the genic regions of 16,014 orthologous genes.

| Sites | *A. thaliana* | *C. rubella* |
| --- | --- | --- |
| total number of sites | 39,275,210 | 40,936,262 |
| number of CpG sites | 2181694 | 2198892 |
| number of non-CpG sites | 37093516 | 38737370 |
| total number of SNPs | 1139630 | 456106 |
| number of SNPs at CpG sites | 211093 | 84147 |
| number of SNPs at non-CpG sites | 928537 | 371959 |
| ratio of SNPs in the CpG region | 0.097 | 0.038 |
| ratio of SNPs in the non-CpG region | 0.025 | 0.010 |
| percentage of the CpG region versus  the whole genic region | 5.6% | 5.4% |

Table S6. Simulation results for different demographic models.

| Model | shSNP >0 | shSNP >1 | shSNP >1 & allelic tree |
| --- | --- | --- | --- |
| M1 | 15,519 | 240 | 0 |
|  |  |  |  |
| M2 | 15,536 | 174 | 0 |
|  |  |  |  |

Table S7. Number of homologous genes for each of the confirmed genes with trans-species polymorphism signals in green plants.

| Species | AT1G35220 | AT2G16570 | AT4G29360 | AT5G38460 | AT5G44000 |
| --- | --- | --- | --- | --- | --- |
| *Arabidopsis lyrata* | 4 | 8 | 6 | 1 | 7 |
| *Arabidopsis thaliana* | 4 | 7 | 6 | 1 | 4 |
| *Capsella rubella* | 4 | 8 | 7 | 1 | 4 |
| *Oryza sativa* | 6 | 9 | 8 | 1 | 2 |
| *Populus trichocarpa* | 7 | 9 | 7 | 2 | 2 |
| *Sorghum bicolor* | 5 | 7 | 6 | 1 | 2 |
| *Zea mays* | 7 | 13 | 6 | 1 | 9 |
| *Amborella trichopoda* | 4 | 4 | 3 | 1 | 3 |
| *Selaginella moellendorffii* | 5 | 6 | 18 | 1 | 6 |
| *Physcomitrella patens* | 6 | 8 | 21 | 1 | 2 |
| *Chlamydomonas reinhardtii* | 4 | 3 | 0 | 1 | 3 |

Table S10. Correlation between the structure and allelic type in 80 *A. thaliana* samples for each of the five genes under balancing selection.

| **AT1G35220** | TSP-1 |  | allele 1 | allele 2 |
| --- | --- | --- | --- | --- |
|  |  | structure 1 | 30 | 30 |
|  |  | structure 2 | 5 | 15 |
|  |  | FDR corrected *P* = 0.2268 | | |
|  |  |  |  |  |
|  | TSP-2 |  | allele 1 | allelic 2 |
|  |  | structure 1 | 30 | 30 |
|  |  | structure 2 | 5 | 15 |
|  |  | FDR corrected *P* = 0.2268 | | |
|  |  |  |  |  |
| **AT2G16570** | TSP-3 |  | allele 1 | allelic 2 |
|  |  | structure 1 | 13 | 47 |
|  |  | structure 2 | 3 | 17 |
|  |  | FDR corrected *P* = 0.8299 | | |
|  |  |  |  |  |
|  | TSP-4 |  | allele 1 | allelic 2 |
|  |  | structure 1 | 12 | 48 |
|  |  | structure 2 | 3 | 17 |
|  |  | FDR corrected *P* = 0.8686 | | |
|  |  |  |  |  |
| **AT4G29360** | TSP-5 |  | allele 1 | allelic 2 |
|  |  | structure 1 | 5 | 55 |
|  |  | structure 2 | 0 | 20 |
|  |  | FDR corrected *P* = 0.5296 | | |
|  |  |  |  |  |
|  | TSP-6 |  | allele 1 | allelic 2 |
|  |  | structure 1 | 5 | 55 |
|  |  | structure 2 | 0 | 20 |
|  |  | FDR corrected *P* = 0.5296 | | |
|  |  |  |  |  |
| **AT5G38460** | TSP-7 |  | allele 1 | allelic 2 |
|  |  | structure 1 | 1 | 59 |
|  |  | structure 2 | 7 | 13 |
|  |  | FDR corrected *P* = 0.0005375 | | |
|  |  |  |  |  |
|  | TSP-8 |  | allele 1 | allelic 2 |
|  |  | structure 1 | 0 | 60 |
|  |  | structure 2 | 7 | 13 |
|  |  | FDR corrected *P* = 0.00001422 | | |
|  |  |  |  |  |
| **AT5G44000** | TSP-9 |  | allele 1 | allelic 2 |
|  |  | structure 1 | 6 | 54 |
|  |  | structure 2 | 0 | 20 |
|  |  | FDR corrected *P* = 0.5296 | | |
|  |  |  |  |  |
|  | TSP-10 |  | allele 1 | allelic 2 |
|  |  | structure 1 | 6 | 54 |
|  |  | structure 2 | 0 | 20 |
|  |  | FDR corrected *P* = 0.5296 | | |
|  |  |  |  |  |
