# Supplementary material for: Long-term balancing selection contributes to adaptation in Arabidopsis and its relatives
Source: Genome Biol. 2017 Nov 15;18:217. doi: 10.1186/s13059-017-1342-8 (PMC5686891; doi:10.1186/s13059-017-1342-8)

**Additional file Figures for**

**Long-term balancing selection contributes to adaptation in *Arabidopsis* and its relatives**

Qiong Wu^1^, Ting-Shen Han^1,2^, Xi Chen^1,2^, Jia-Fu Chen^1,2^, Yu-Pan Zou^1,2^, Zi-Wen Li^1^, Yong-Chao Xu^1,2^, Ya-Long Guo^1,2*^

^1^ State Key Laboratory of Systematic and Evolutionary Botany, Institute of Botany, Chinese Academy of Sciences, Beijing 100093, China

^2^ University of Chinese Academy of Sciences, Beijing 100049, China

* Corresponding author: Ya-Long Guo

State Key Laboratory of Systematic and Evolutionary Botany

Institute of Botany, Chinese Academy of Sciences

Beijing 100093, China

PH +86-62836298; FX +86-62590843

EM yalong.guo@ibcas.ac.cn

Running title: Balancing selection shapes evolution of *Arabidopsis* relatives

Fig. S1 Allelic trees across the two species based on the 100 bp window around the TSP sites for each of the five genes under balancing selection. All *A. thaliana* accessions are colored in red and numbered according to the accessions listed on the 1001 Genomes site (http://1001genomes.org/projects/MPICao2010/index.html); see Table S8 for details. All *C. rubella* accessions are shown in black and numbered according to Table S1.


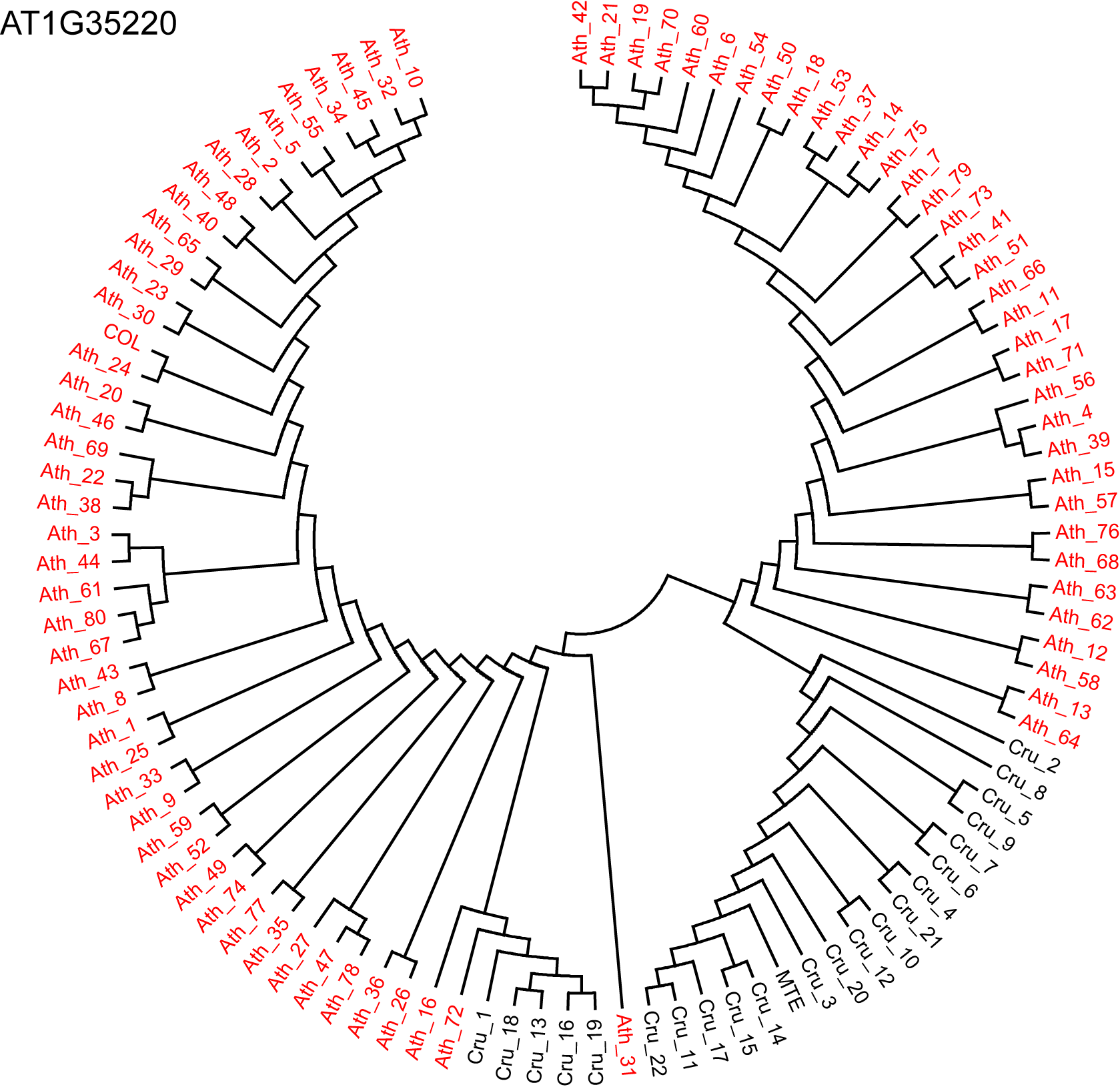


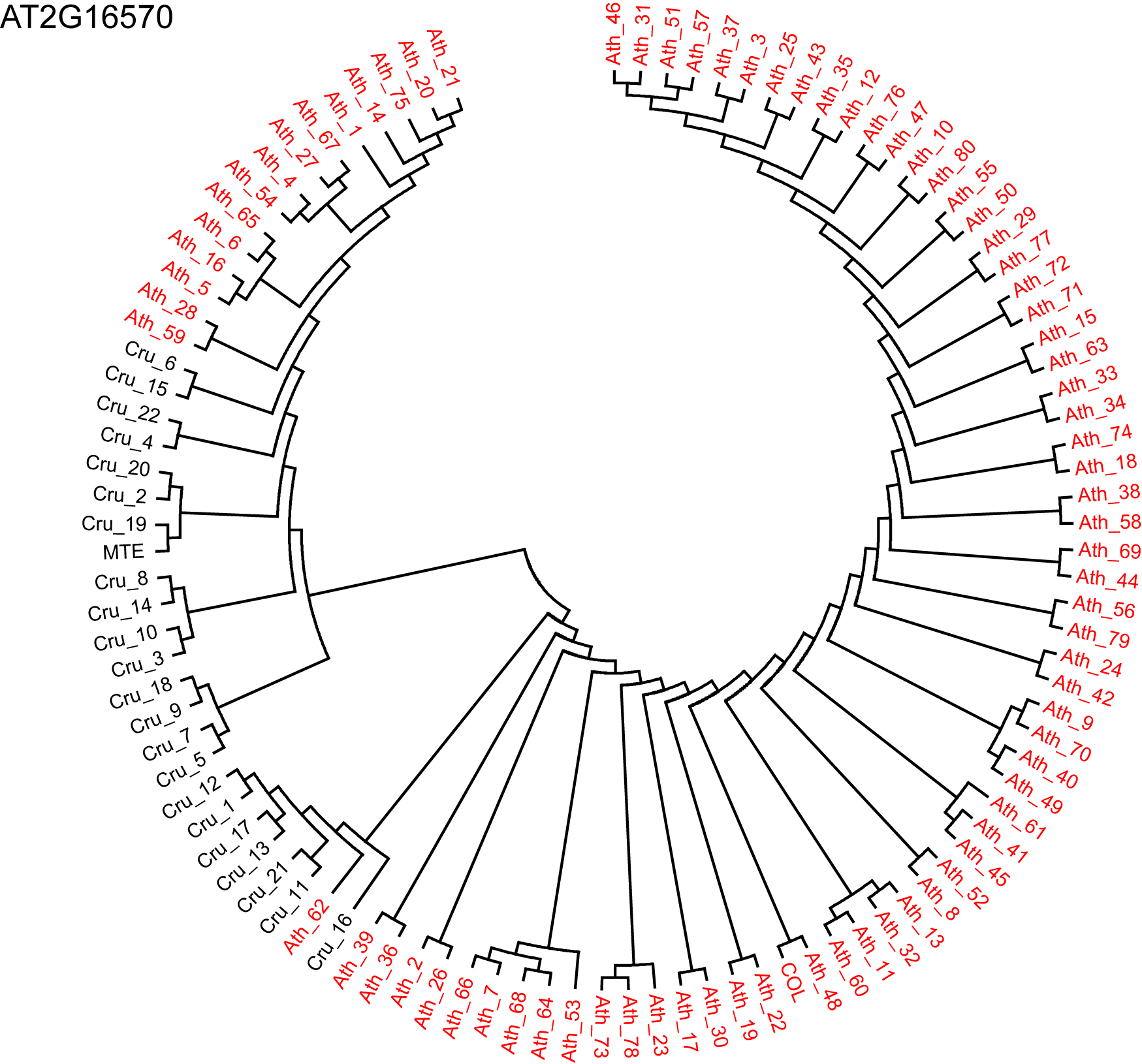


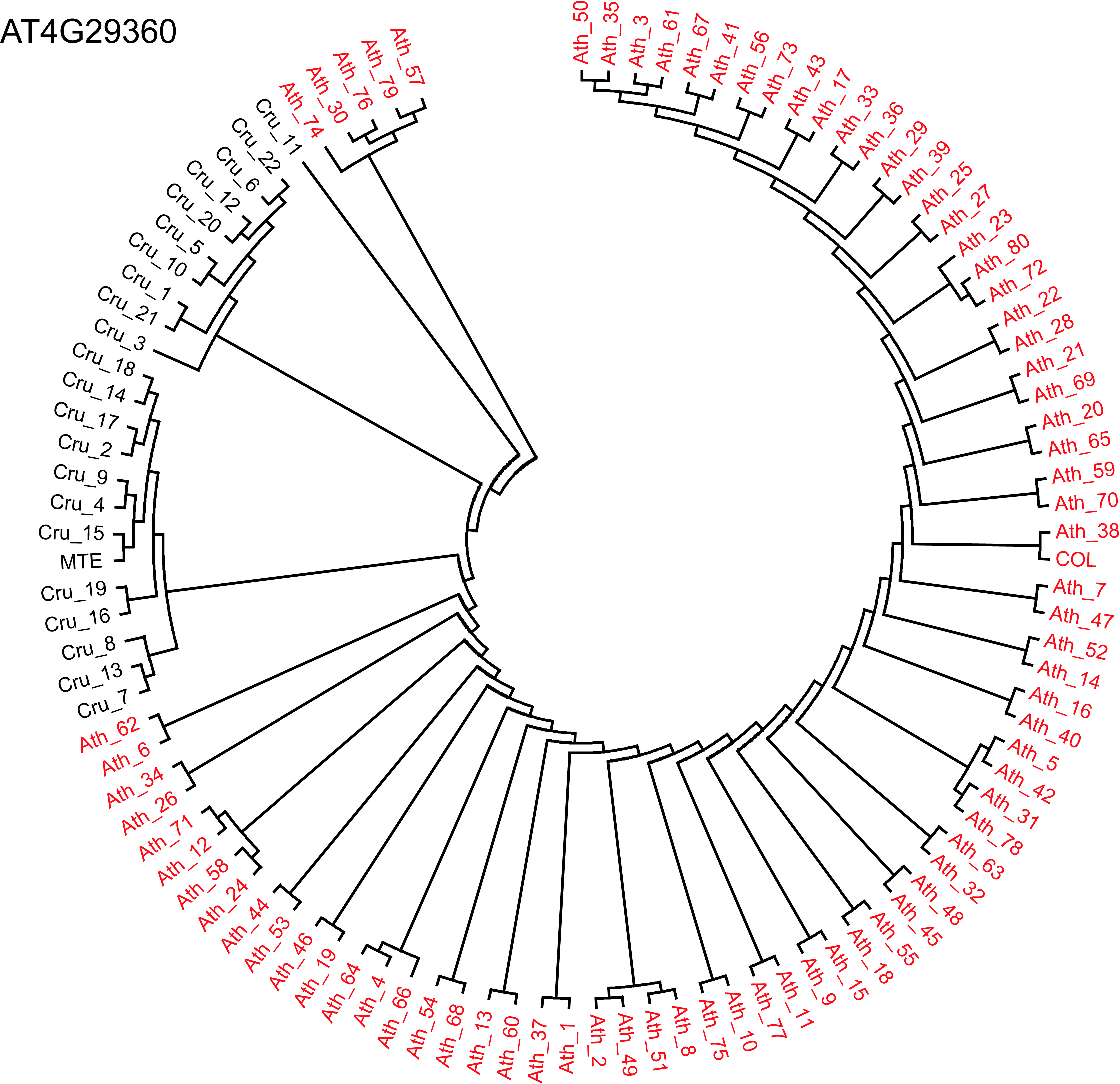


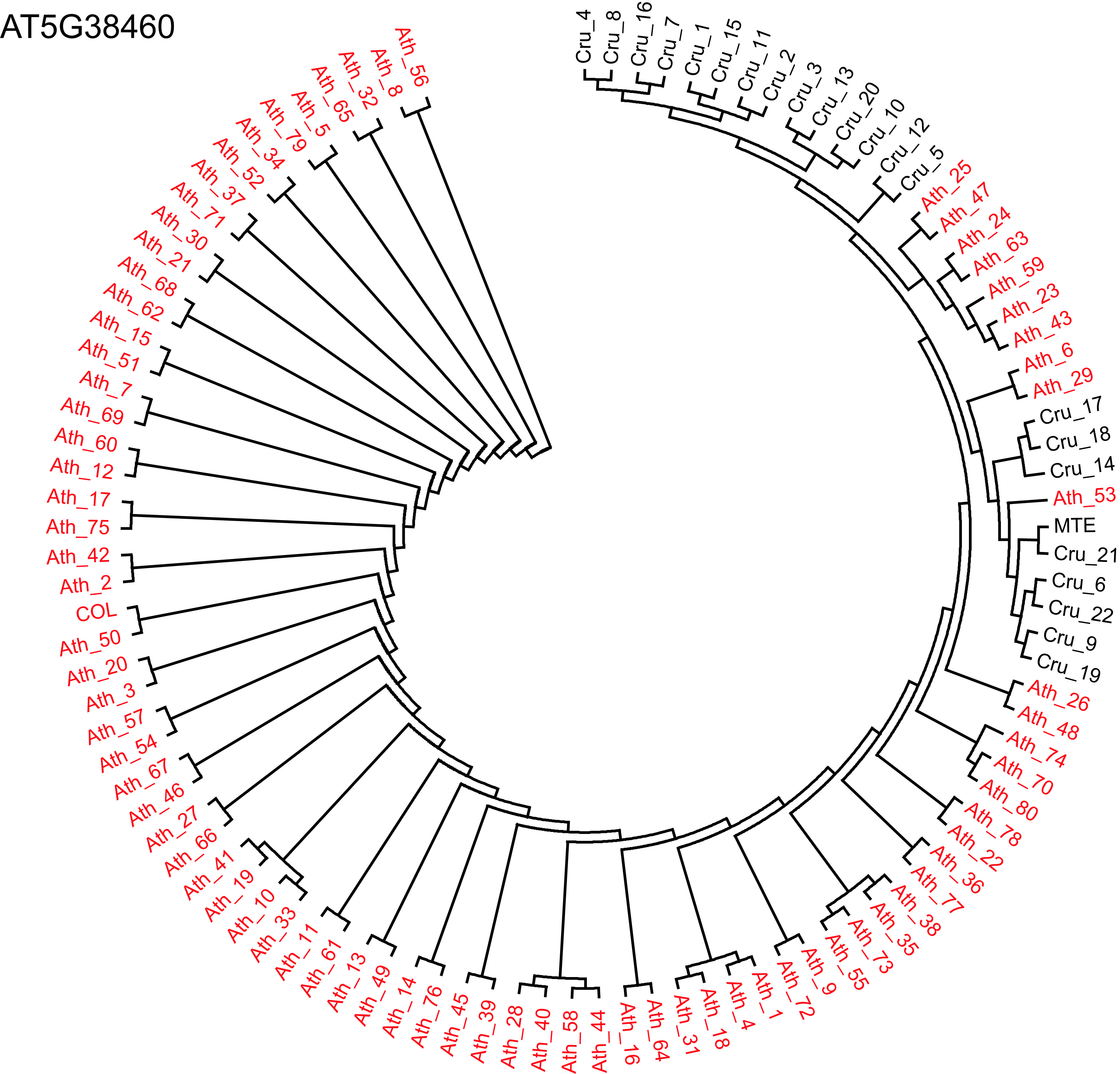


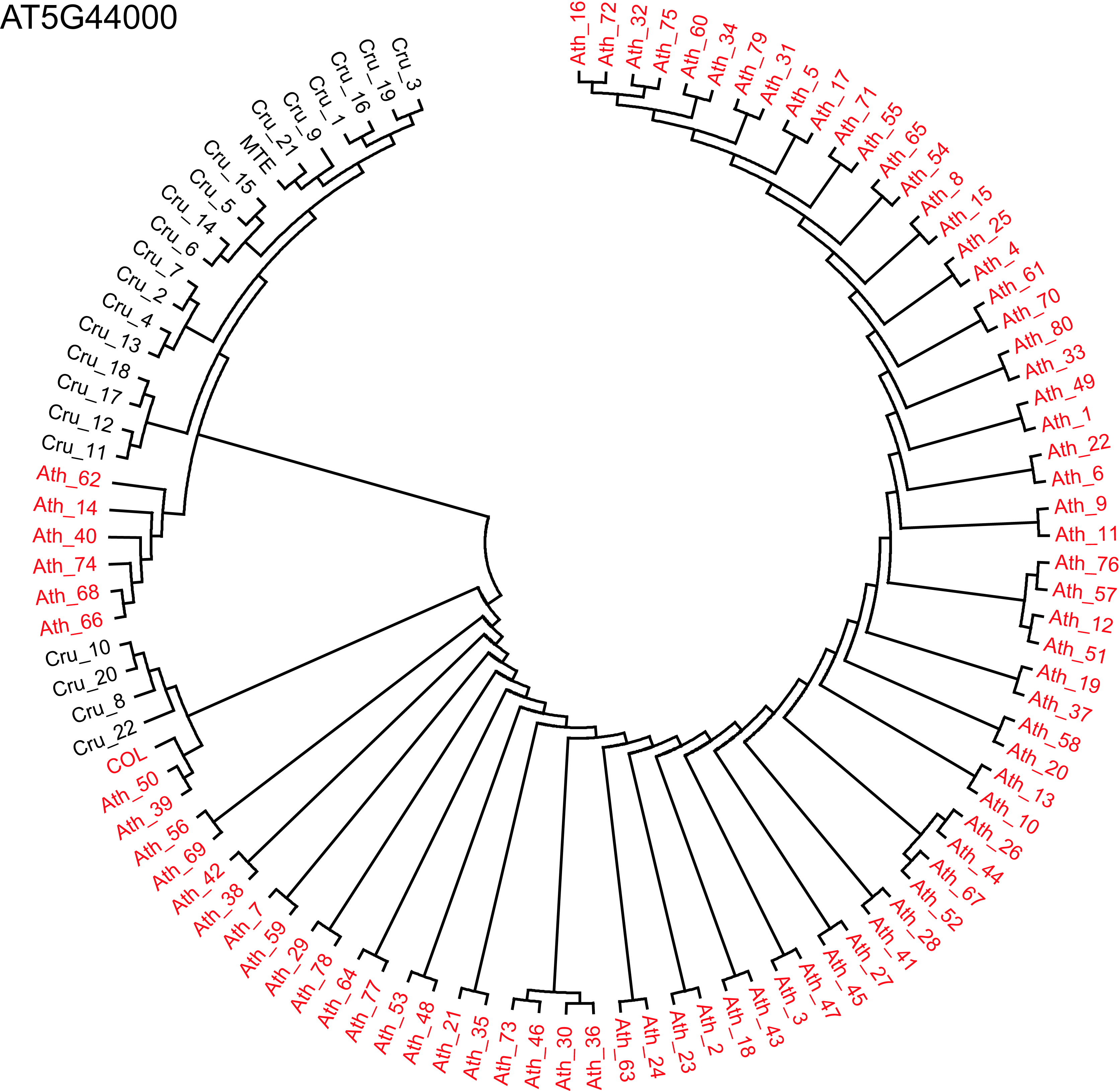


Fig. S2 Distribution of the (A) nucleotide diversity (π) and (B) MAF values of the simulated neutral sequences of 100 bp under the estimated model in each species. Triangles in different colors in (A) indicate the average values for all qualified windows in the five genes. See Table 1 for the details of each site (labeled TSP-1 to TSP-10).


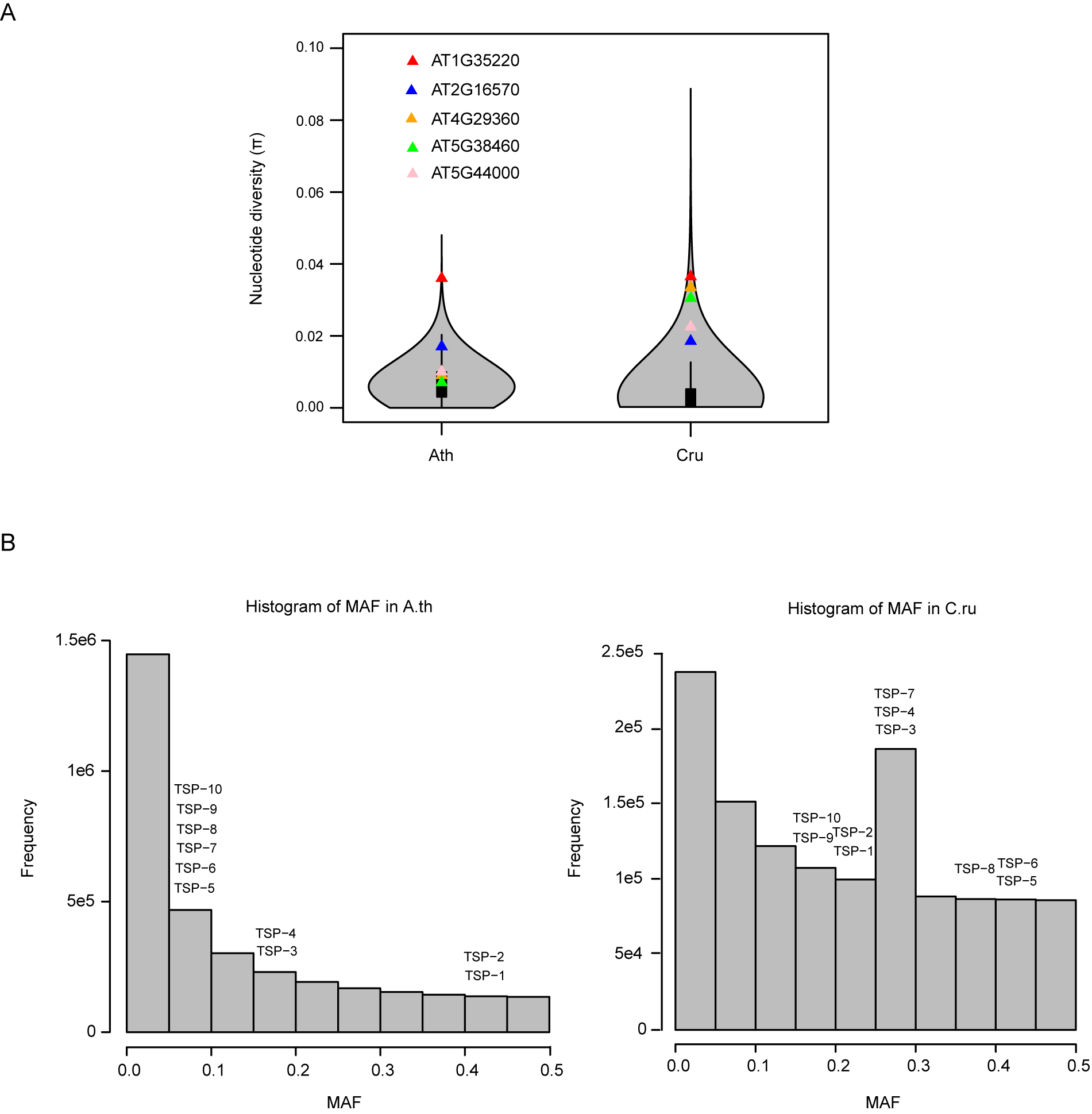


Fig. S3 Cross-validation errors for various numbers of clusters (*K*) in an ADMIXTURE analysis.


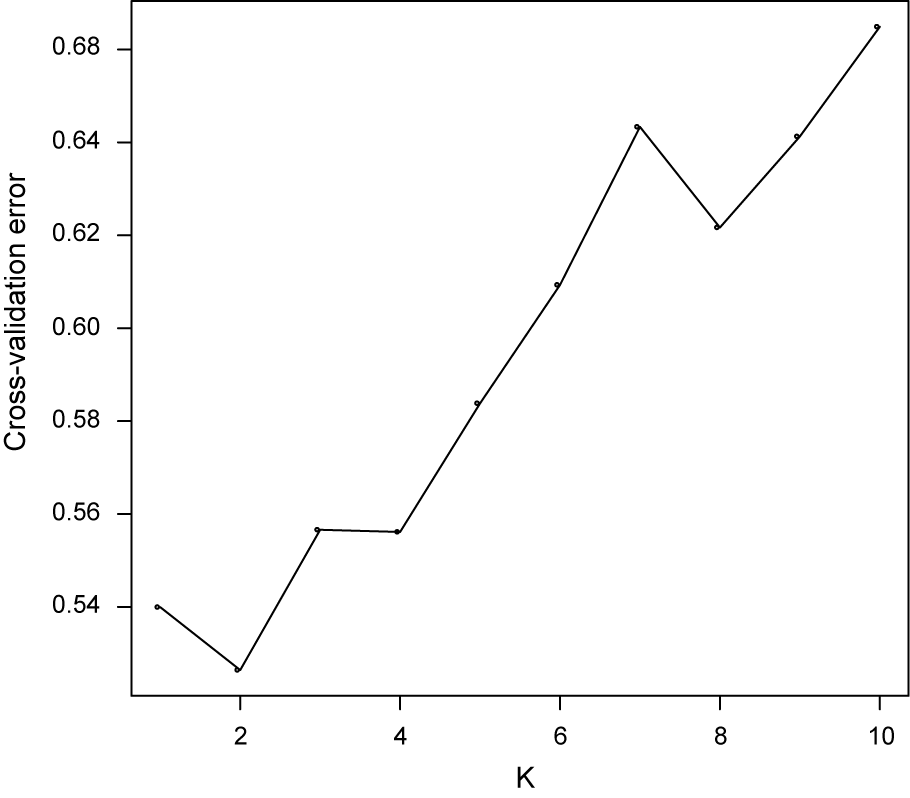


Fig. S4 Geographic distributions of samples of different allelic types for the four genes under long-term balancing selection excluding AT5G38460.

AT1G35220


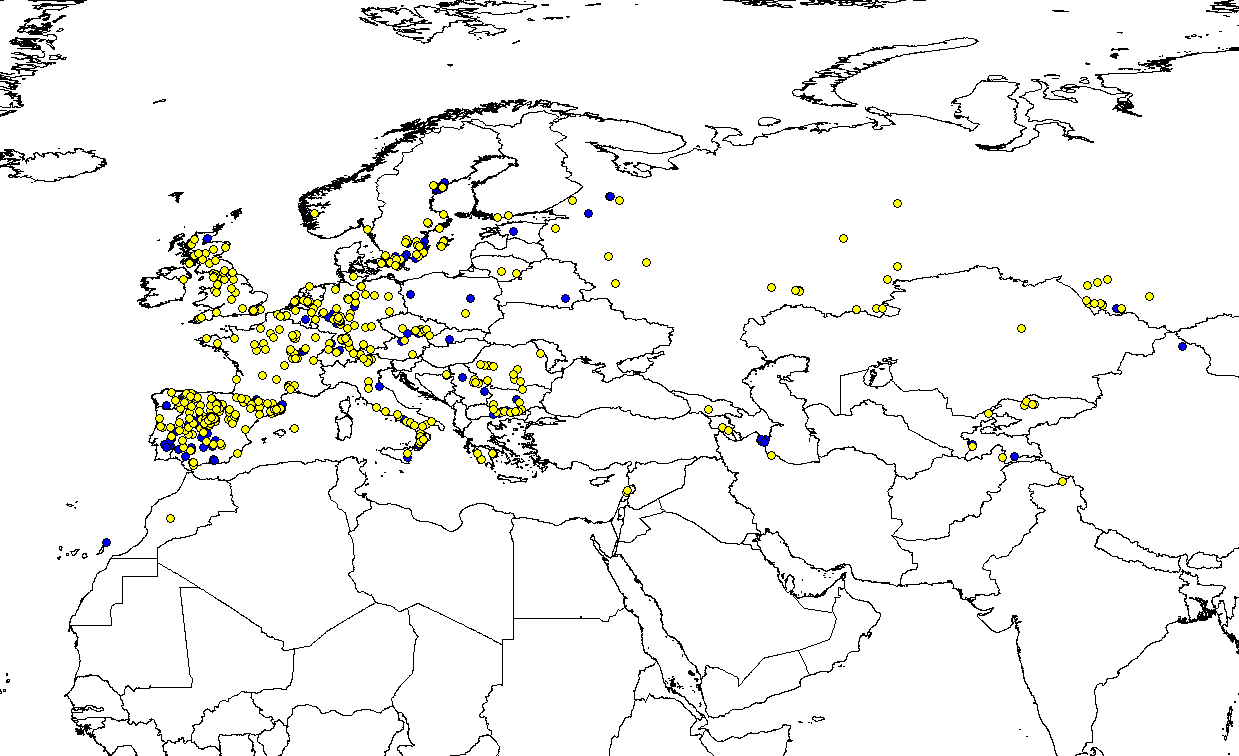


AT2G16570


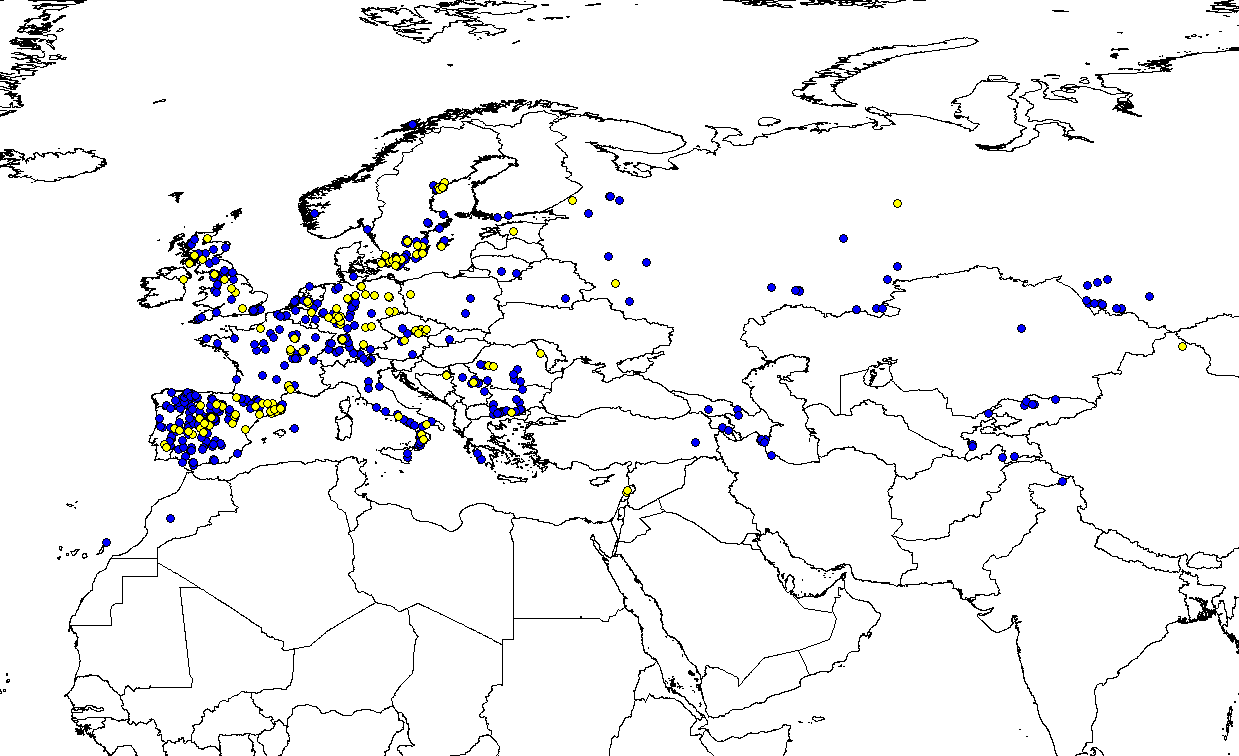


AT3G29460


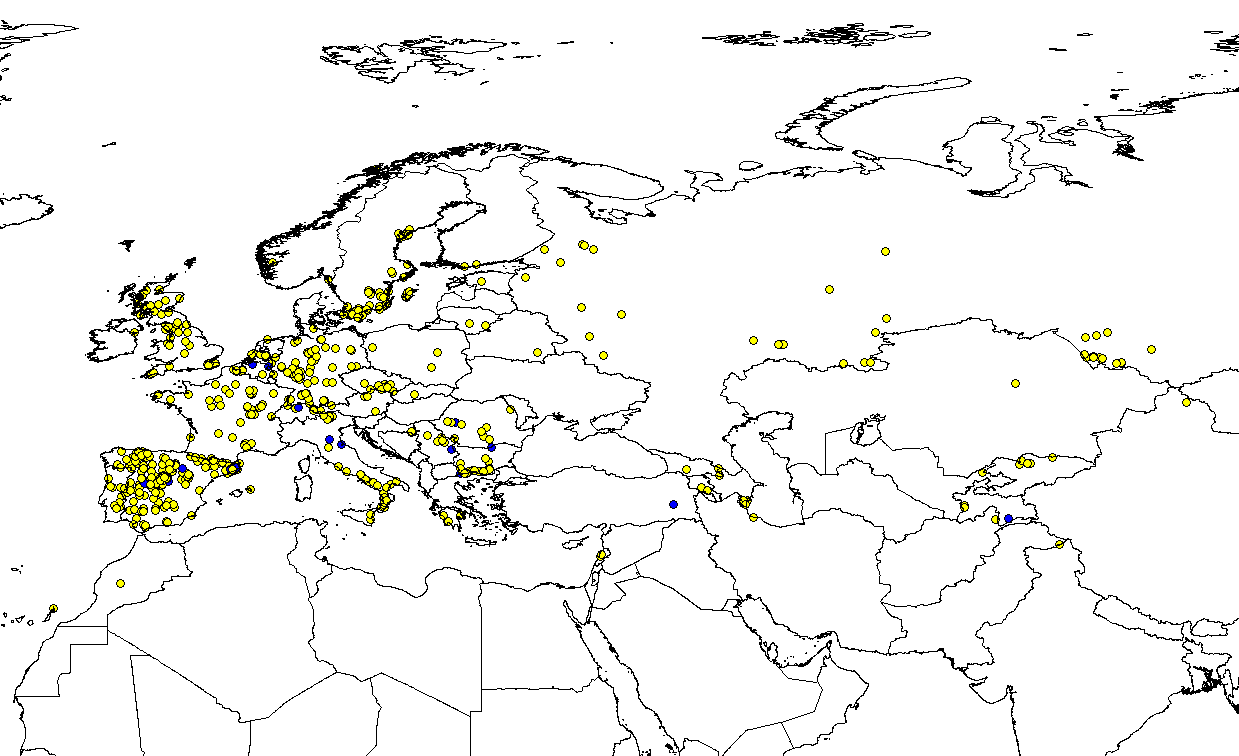


AT5G44000


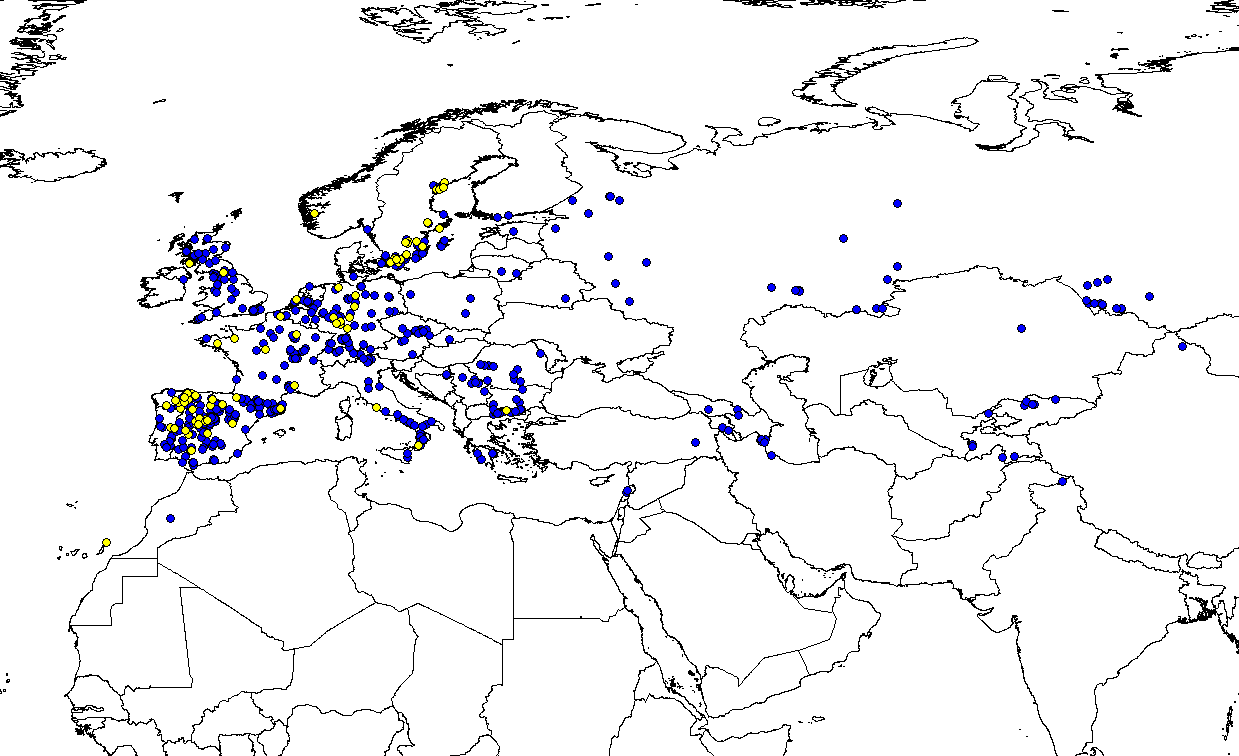

Supplement: Supplementary file 3 — Allelic trees across the two species based on the 100-bp window around the TSP sites for each of the five genes under balancing selection. All A. thaliana accessions are colored in red and numbered according to the accessions listed on the 1001 Genomes site (http://1001genomes.org/projects/MPICao2010/index.html); see Additional file 5: Table S8C for details. All C. rubella accessions are shown in black and numbered according to Additional file 1: Table S1. Figure S2. Distribution of the (A) nucleotide diversity (π) and (B) MAF values of the simulated neutral sequences of 100 bp under the estimated model in each species. Triangles in different colors in (A) indicate the average values for all qualified windows in the five genes. See Table 1 for the details of each site (labeled TSP-1 to TSP-10). Figure S3. Cross-validation errors for various numbers of clusters (K) in an ADMIXTURE analysis. Figure S4. Geographic distributions of samples of different allelic types for the four genes under long-term balancing selection excluding AT5G38460. (DOCX 5905 kb) [file 13059_2017_1342_MOESM3_ESM.docx]
