# Supplementary material for: Long-term balancing selection contributes to adaptation in Arabidopsis and its relatives
Source: Genome Biol. 2017 Nov 15;18:217. doi: 10.1186/s13059-017-1342-8 (PMC5686891; doi:10.1186/s13059-017-1342-8)
Supplement: Supplementary file 4 — Text S1. Coalescence-based simulation to support the candidate TSPs. (DOCX 45 kb) [file 13059_2017_1342_MOESM4_ESM.docx]

**Additional file Text for**

**Long-term balancing selection contributes to adaptation in *Arabidopsis* and its relatives**

Qiong Wu^1^, Ting-Shen Han^1,2^, Xi Chen^1,2^, Jia-Fu Chen^1,2^, Yu-Pan Zou^1,2^, Zi-Wen Li^1^, Yong-Chao Xu^1,2^, Ya-Long Guo^1,2*^

^1^ State Key Laboratory of Systematic and Evolutionary Botany, Institute of Botany, Chinese Academy of Sciences, Beijing 100093, China

^2^ University of Chinese Academy of Sciences, Beijing 100049, China

* Corresponding author: Ya-Long Guo

State Key Laboratory of Systematic and Evolutionary Botany

Institute of Botany, Chinese Academy of Sciences

Beijing 100093, China

PH +86-62836298; FX +86-62590843

EM yalong.guo@ibcas.ac.cn

Running title: Balancing selection shapes evolution of *Arabidopsis* relatives

**Text S1. Coalescence-based simulation to support the candidate TSPs**

To see how often the candidate TSP regions detected in this study are expected to occur by recurrent mutations or gene flow under neutral evolution, but are not maintained by balancing selection, we simulated neutral sequences using fastsimcoal2 [[1](#_ENREF_1)] based on the best estimates under both Model M1 and M2. We simulated 100 bp windows matching the real sizes of 81 *A. thaliana* accessions and 23 *C. rubella* accessions 1,000,000 times.

Since the heterogeneity of mutation rates can greatly affect the rate of recurrent mutations, and notably, CpG sites in mammals have a much higher mutation rate than non-CpG sites [[2](#_ENREF_2)], we modeled CpG and non-CpG sites separately in our simulation. We first calculated the mutation rates of these two types of sites (assuming each type has the same mutation rate) by counting the number of CpG (non-CpG) sites, and the number of polymorphisms at these two types of sites, respectively (see Table S5 for the statistics). Finally, we found that approximately 6% of the genome is CpG sites, and the mutation rate at CpG sites is about 4-fold greater than that at non-CpG sites, supported by both *A. thaliana* and *C. rubella* data. Taking the general mutation rate as 7 × 10^-9^/bp/gen, the following equation can be solved:

$4\mu\times0.06+\mu\times0.94=7 \times{10}^{-9}$.

We thereby obtained a mutation rate $4\mu$for CpG regions of 2.4 × 10^-8^/bp/gen, and a mutation rate $\mu$for non-CpG regions of 5.9 × 10^-9^/bp/gen.

For each 100 bp window, we simulated 94 non-CpG sites and 6 CpG sites, separately, using their respective mutation rates. Then, we counted the number of cases with shared SNPs (shTSP >0), and two or more shared SNPs (shTSP >1), and finally the number of sequences with two or more shared SNPs and clustering by allele (shSNP >1 & allelic tree). Details of the results are provided in Additional File 1: Table S6.

These results suggest that neutral shared SNPs can occur (by either recurrent mutation or gene flow), but the sequences cannot evolve neutrally following an allelic tree, which helps validate that the five genes we found are not consistent with neutral evolution and are real TSPs under balancing selection.

**References**

1. Excoffier L, Dupanloup I, Huerta-Sanchez E, Sousa VC, Foll M: **Robust demographic inference from genomic and SNP data**. *PLoS Genet* 2013, **9**(10):e1003905.

2. Hodgkinson A, Eyre-Walker A: **Variation in the mutation rate across mammalian genomes**. *Nat Rev Genet* 2011, **12**(11):756-766.
